# SNORA28 Promotes Proliferation and Radioresistance in Colorectal Cancer Cells through the STAT3 Pathway by Increasing H3K9 Acetylation in the LIFR Promoter

**DOI:** 10.1002/advs.202405332

**Published:** 2024-06-26

**Authors:** Xin Liu, Hong Zhang, Ying Fan, Dan Cai, Ridan Lei, Qi Wang, Yaqiong Li, Liping Shen, Yongqing Gu, Qingtong Zhang, Zhenhua Qi, Zhidong Wang

**Affiliations:** ^1^ Department of Radiobiology Beijing Key Laboratory for Radiobiology Beijing Institute of Radiation Medicine Beijing 100850 China; ^2^ Graduate Collaborative Training Base of Academy of Military Sciences Hengyang Medical School University of South China Hengyang Hunan 421001 China; ^3^ Department of Epidemiology and Health Statistics Xiangya School of Public Health Central South University Changsha Hunan 410078 China; ^4^ Department of Colorectal Surgery Liaoning Cancer Hospital & Institute Cancer Hospital of China Medical University Cancer Hospital of Dalian University of Technology Shenyang 110042 China

**Keywords:** colorectal cancer, histone acetylation, LIFR, radiotherapy, SNORA28, STAT3 pathway

## Abstract

Radiotherapy is essential for treating colorectal cancer (CRC), especially in advanced rectal cancer. However, the low radiosensitivity of CRC cells greatly limits radiotherapy efficacy. Small nucleolar RNAs (snoRNAs) are a class of noncoding RNA that primarily direct post‐transcriptional modifications of ribosomal RNAs (rRNAs), small nuclear RNAs (snRNAs), and other cellular RNAs. While snoRNAs are involved in tumor progression and chemoresistance, their association with radiosensitivity remains largely unknown. Herein, SNORA28 is shown highly expressed in CRC and is positively associated with poor prognosis. Furthermore, SNORA28 overexpression enhances the growth and radioresistance of CRC cells in vitro and in vivo. Mechanistically, SNORA28 acts as a molecular decoy that recruits bromodomain‐containing protein 4 (BRD4), which increases the level of H3K9 acetylation at the LIFR promoter region. This stimulates LIFR transcription, which in turn triggers the JAK1/STAT3 pathway, enhancing the proliferation and radioresistance of CRC cells. Overall, these results highlight the ability of snoRNAs to regulate radiosensitivity in tumor cells and affect histone acetylation modification in the promoter region of target genes, thus broadening the current knowledge of snoRNA biological functions and the mechanism underlying target gene regulation.

## Graphical abstract

SNORA28 is highly expressed in CRC and is positively associated with poor prognosis. SNORA28 acts as a molecular decoy that recruits BRD4, which increases the level of H3K9ac at the LIFR promoter region. This stimulates LIFR transcription, which in turn triggers the JAK1/STAT3 pathway, enhancing the proliferation and radioresistance of CRC cells.

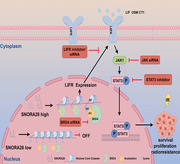

## 1 Introduction

Colorectal cancer (CRC) is the third most common malignancy and the second leading cause of cancer‐related deaths worldwide.^[^ ^1^ ^]^ Surgery and neoadjuvant chemoradiotherapy comprise the primary treatments for CRC. Radiotherapy is a standard method of preoperative treatment in locally advanced rectal cancer.^[^ ^2^ ^]^ Radiotherapy improves surgical efficacy, reduces local recurrence, and improves the survival rate of patients with cancer. However, only 15%–20% of patients with CRC achieve complete remission after radiotherapy.^[^ ^3^ ^]^ Given that many patients with CRC show low sensitivity or even complete resistance to radiotherapy, elucidating the molecular mechanism underlying CRC radioresistance is crucial for identifying more effective therapeutic targets and improving radiotherapy efficacy.

Small nucleolar RNAs (snoRNAs), first identified in 1960, are a class of conserved non‐coding RNA molecules, 60–300 nucleic acids long, localized primarily within the nucleus.^[^ ^4^ ^]^ Based on the differences in conserved sequence elements and structures, they are primarily divided into H/ACA box snoRNAs and C/D box snoRNAs,^[^ ^5^ ^]^ which play a crucial role in splicing and post‐transcriptional modification of ribosomes.^[^ ^6^ ^]^ The development of high‐throughput sequencing and powerful analysis tools have enabled the identification of several snoRNAs, which are involved in a variety of physiological and pathological processes, such as in neurological disorders,^[^ ^7^ ^]^ cardiovascular diseases,^[^ ^8^ ^]^ and cancer.^[^ ^9^ ^]^ While the dysregulation of snoRNAs (e.g., SNORD1C,^[^ ^10^ ^]^ ACA11,^[^ ^11^ ^]^ and SNORA23 ^[^ ^12^ ^]^) has been shown to affect tumor progression and chemoresistance, their influence on radiosensitivity has rarely been reported. SnoRNAs are involved in tumor cell survival, apoptosis, and cell cycle by regulating the signal transduction cascades.^[^ ^13^ ^]^ Overexpressing specific snoRNAs in tumor cells sensitive to doxorubicin, a DNA‐damaging agent with a similar mechanism of action to radiotherapy, was shown to enhance doxorubicin resistance.^[^ ^14^ ^]^ These findings provide important insights into the involvement of snoRNAs in the regulation of tumor cells' radiosensitivity.

The signal transducer and activator of the transcription 3 (STAT3) pathway plays an essential role in cellular proliferation, survival, apoptosis, angiogenesis, and immune response.^[^ ^15^ ^]^ Phosphorylation of STAT3 at Tyr705 is considered a hallmark of STAT3 pathway activation,^[^ ^16^ ^]^ and its hyperactivation, which has been well documented in approximately 70% of cancers,^[^ ^17^ ^]^ enhances radioresistance in various tumors,^[^ ^18^ ^]^ including colorectal,^[^ ^19^ ^]^ breast,^[^ ^20^ ^]^ and glioma.^[^ ^21^ ^]^ In mouse models, genetic dysregulation is accompanied by the activation of STAT3 signaling, which accentuates the formation of colorectal adenomas.^[^ ^22^ ^]^ Additionally, intrinsic activation of STAT3 contributes to the persistent growth of CRC cells after irradiation through anti‐apoptotic and pro‐survival mechanisms.^[^ ^19^ ^]^ As STAT3 plays a major role in the tumorigenesis and radioresistance of tumor cells, the development of targeted therapeutics for STAT3 inhibition is considered a promising approach for cancer treatment.^[^ ^23^ ^]^ Although no STAT3 inhibitors are currently on the market, some (e.g., Napabucasin, OPB‐31121, and DSP‐0337) have successfully entered clinical trials, yielding encouraging results.^[^ ^24^ ^]^

In light of the limited information on snoRNAs in the context of radioresistance and the central role of STAT3 in tumorigenesis and treatment response, we aimed to assess the regulatory effects of SNORA28 on the STAT3 pathway. Further, we explored the role and molecular mechanism of SNORA28 in CRC radioresistance to explore new avenues for overcoming CRC radioresistance.

## 2 Results

### 2.1 SNORA28 is Highly Expressed in CRC and is Associated with a Poor Prognosis in Patients

**Figure 1:**
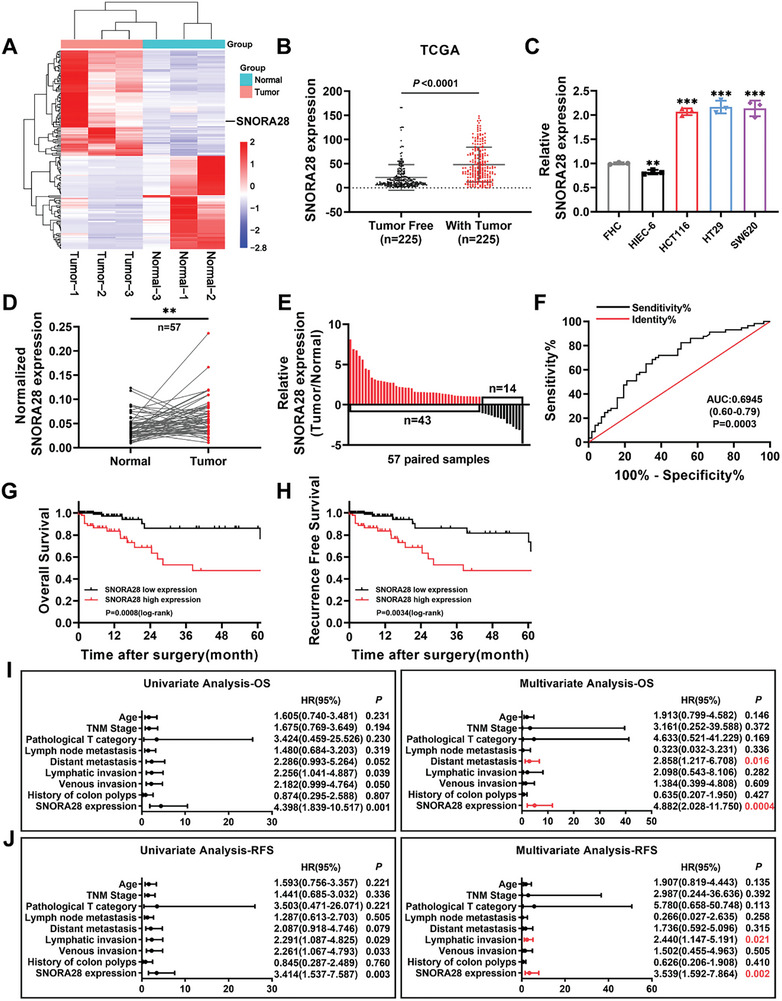
SNORA28 is highly expressed and predicts poor prognosis in patients with CRC. A) Hierarchical clustering analysis of differentially expressed snoRNA in 3 pairs CRC tissues and para‐cancer tissues. B) TCGA database analysis of SNORA28 expression in patients with CRC versus tumor‐free patients (*n* = 225). C) qRT‐PCR analysis of the relative expression of SNORA28 in the three human CRC cell lines and two normal human epithelial cell lines. D–E) qRT‐PCR analysis of the relative SNORA28 expression in 57 pairs of CRC tissues and matched para‐cancer tissues. F) The Receiver Operating Characteristic (ROC) curve of SNORA28 for distinguishing tissues with CRC and normal controls (*n* = 57). G–H) Kaplan‐Meier analyses of overall survival (OS) (G) and recurrence‐free survival (RFS) (H) for CRC patients (*n* = 335) according to SNORA28 expression levels. I–J) Univariate and multivariate analyses of OS (I) and RFS (J) in patients with CRC (*n* = 335). ^**^ *p* < 0.01, ^***^ *p* < 0.001.

Differentially expressed snoRNAs were analyzed in tumor tissues and adjacent normal tissues from three patients with CRC using the nrStar Human snoRNA PCR chip. A total of 147 co‐regulated snoRNAs were identified, among which 78 and 69 were upregulated and downregulated, respectively (**Figure** **1**A; Table S1, Supporting Information). SNORA28 was found to be highly expressed in the tumor tissues of all three patients, and The Cancer Genome Atlas (TCGA) database analysis showed that SNORA28 was significantly upregulated in CRC patients with tumors when compared to in those without (Figure 1B). Furthermore, the SNORic database revealed high SNORA28 expression in various tumor types (Figure S1, Supporting Information). Compared with that in normal human colorectal epithelial cells (FHC) and human small intestinal epithelial cells (HIEC‐6), SNORA28 expression was significantly elevated in three CRC cell lines (HCT116, HT29, and SW620) (Figure 1C). Data from 57 pairs of CRC samples showed that SNORA28 was highly expressed in 75% (43/57) of the CRC tissues (Figure 1D,E). Receiver Operating Characteristic (ROC) curve analysis of 57 samples showed that SNORA28 distinguished CRC tissues from normal colon epithelial tissues (Figure 1F). Analysis of TCGA data for assessing the relevance of SNORA28 expression to clinicopathological factors in patients with CRC showed that SNORA28 expression was significantly correlated with a history of colon polyps, lymphatic invasion, and venous invasion (Table 1). Furthermore, among patients with CRC, those with high SNORA28 expression exhibited lower overall survival (OS) and shorter recurrence‐free survival (RFS) than those with low SNORA28 expression (Figure 1G,H). Univariate and multivariate Cox proportional hazards models showed that SNORA28 could be an independent prognostic factor for OS and RFS in patients with CRC (Figure 1I,J).

**Table 1:** Correlations between SNORA28 expression and clinicopathological parameters in patients with CRC.

			SNORA28 Expression			
Characteristics		Case	Low	High	χ^2^	*p*
Sex					1.315	0.251
	Female	166	88	78		
	Male	169	79	90		
Age(year)					2.264	0.132
	≤70	195	104	91		
	>70	140	63	77		
TNM stage (AJCC)					0.239	0.625
	Stage I/II	191	93	98		
	Stage III/IV	144	74	70		
Pathological T category					0.652	0.419
	T1/T2	64	29	35		
	T3/T4	271	138	133		
Lymph node metastasis					0.674	0.412
	Negative	194	93	101		
	Positive	141	74	67		
Distant metastasis					1.698	0.193
	Negative	280	144	136		
	Positive	55	23	32		
Lymphatic invasion					8.246	0.004
	Negative	205	115	90		
	Positive	130	52	78		
Venous invasion					3.877	0.049
	Negative	249	132	117		
	Positive	86	35	51		
History of colon polyps					13.063	0.000
	Negative	230	130	100		
	Positive	105	37	68		

Abbreviations: AJCC, American Joint Committee on Cancer.

The cutoff threshold of SNORA28 expression is the median value in all patients in this cohort.

### 2.2 SNORA28 Promotes CRC Cell Proliferation and Radioresistance

**Figure 2:**
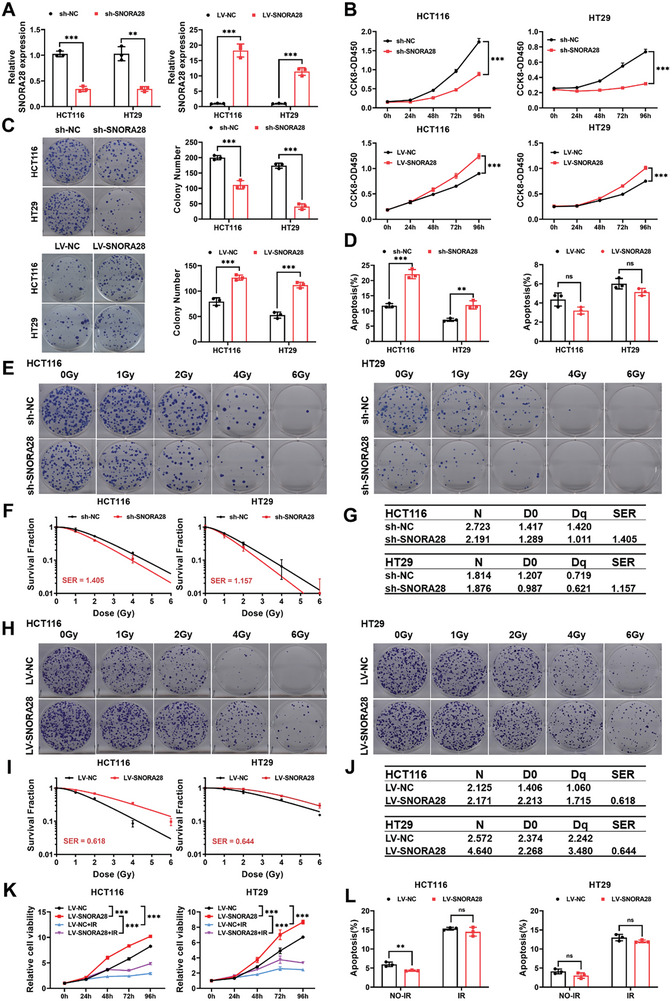
SNORA28 promotes CRC cell proliferation and radioresistance in vitro. A) SNORA28 levels in CRC cells transfected with sh‐SNORA28 and infected with lentiviruses LV‐SNORA28. B–D) CCK8 (B), colony formation (C), and flow cytometry (D) analyses were performed to evaluate CRC cell proliferation (*n* = 5), survival ability (*n* = 3), and apoptotic rates (*n* = 3) after SNORA28 knockdown or overexpression. E) Representative images of colonies that survived irradiation in CRC cell colonies after SNORA28 knockdown. F) Survival curves for irradiated CRC cells after SNORA28 knockdown. G) The multi‐target single‐hit model analysis of *N*, *D_0_*, *Dq*, and SER values in CRC cells after SNORA28 knockdown. H) Representative images of colonies of SNORA28 overexpressing CRC cell colonies that survived irradiation (*n* = 3). I) Survival curves for irradiation in CRC cells with SNORA28 overexpression. J) The multi‐target single‐hit model analysis of the values of *N*, *D_0_*, *Dq*, and SER in CRC cells with SNORA28 overexpression. K, L) CCK8 (K) and flow cytometry (L) analyses of proliferation (*n* = 5) and apoptotic rates (*n* = 3) in CRC cells treated with 8 Gy irradiation under SNORA28 overexpression. Data are represented as the means ± standard deviation (SD). ^**^ *p* < 0.01; ^***^ *p* < 0.001; ns, no significance.

To further investigate SNORA28 function in CRC, the RNA‐fold web server was used to predict the secondary structure of SNORA28 (Figure S2, Supporting Information). HCT116 and HT29 cells were transfected with shRNA sequences to knock down SNORA28 or infected with lentiviruses to overexpress SNORA28. The efficiency of SNORA28 knockdown and overexpression was determined using RT‐qPCR (**Figure** **2**A). SNORA28 knockdown significantly inhibited CRC cell proliferation and survival. Conversely, SNORA28 overexpression promoted CRC cell proliferation and survival (Figure 2B,C). Flow cytometry showed that SNORA28 knockdown significantly induced apoptosis, whereas SNORA28 overexpression only slightly suppressed it (Figure 2D; Figure S3A,B, Supporting Information). The expression of apoptosis‐related proteins (PARP and Bcl‐2) was regulated by SNORA28 (Figure S3C,D, Supporting Information). Meanwhile, the colony formation assays showed that SNORA28 knockdown significantly increased radiosensitivity in CRC cells, with sensitization‐enhancement ratios (SERs) of 1.405 and 1.157, respectively (Figure 2E–G). In contrast, SNORA28 overexpression produced a pronounced enhancement in the number of surviving colonies and radioresistance of HCT116 and HT29 cells, with SERs of 0.618 and 0.644, respectively (Figure 2H–J). Additionally, overexpression of SNORA28 weakened the inhibitory effect of irradiation on CRC cell proliferation (Figure 2K). Notably, LV‐SNORA28+IR group exhibited increased Bcl‐2 expression and lower Cleaved PARP levels when compared with that in the LV‐NC+IR group, yet without a significant effect on apoptosis after irradiation (Figure 2L; Figure S3E,F, Supporting Information).

### 2.3 SNORA28 Enhances CRC Cell Proliferation and Radioresistance by Activating the JAK1/STAT3 Signaling Pathway

**Figure 3:**
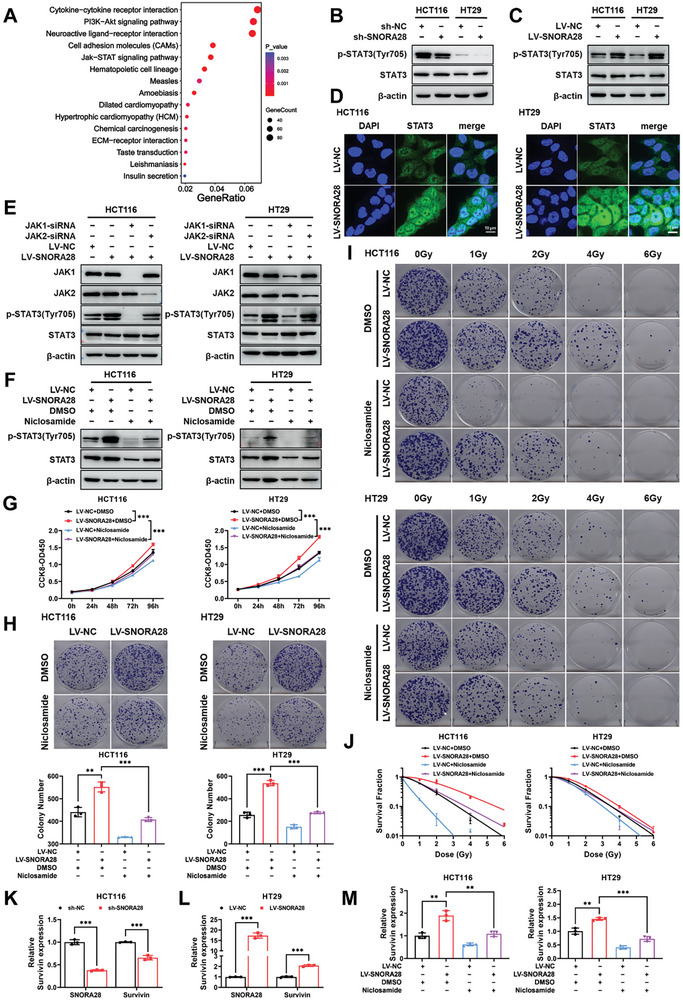
JAK1/STAT3 pathway mediates the biological effects of SNORA28 overexpression in CRC cells. A) The top 15 enriched pathways under SNORA28 overexpression were analyzed via KEGG. B, C) Western blotting analysis of the STAT3 pathway proteins in CRC cells after SNORA28 knockdown (B) and overexpression (C). D) Subcellular localization of STAT3 was analyzed via immunofluorescence staining. E) Western blotting analysis of the indicated proteins in CRC cells treated with JAK1 siRNA and JAK2 siRNA for 48 h in the presence of SNORA28 overexpression. F) Western blotting analysis of the indicated proteins in CRC cells treated with Niclosamide under SNORA28 overexpression. G–H) CCK8 (G) and colony formation (H) analyses of the proliferation (*n* = 5) and survival ability (*n* = 3) of SNORA28 overexpressing CRC cells treated with Niclosamide. I–J) Colony formation assays (I) and survival curves (J) reflect the radiosensitivity in SNORA28 overexpressing CRC cells treated with Niclosamide (*n* = 3). K–L) The expression of downstream target gene Survivin of STAT3 pathway in CRC cells after SNORA28 knockdown (K) and overexpression (L) was analyzed via qRT‐PCR. M) The expression level of Survivin after STAT3 pathway suppression in the presence of SNORA28 overexpression. All CRC cells that stably overexpressed SNORA28 were treated with 2.5 µM Niclosamide for 12 h. Data are represented as the means ± SD. ^**^ *p* < 0.01, ^***^ *p* < 0.001.

To elucidate the underlying molecular mechanism through which SNORA28 enhances CRC cell proliferation and radioresistance, RNA‐sequencing was performed on HCT116 cells infected with LV‐NC or LV‐SNORA28. Differentially expressed genes were screened with fold change > ±1.5 and *p *< 0.05 as the threshold. We then performed the Kyoto Encyclopedia of Genes and Genomes (KEGG) signaling pathway enrichment analysis on 1428 differentially expressed genes (Table S2, Supporting Information) that were identified. The data revealed significant enrichment of the PI3K/AKT and JAK/STAT pathways, which are closely related to tumor progression and radioresistance (**Figure** **3**A). Further verification showed that SNORA28 exerted inconsistent regulatory effects on the PI3K/AKT pathway in HCT116 and HT29 cells, which prompted us to exclude the involvement of PI3K/AKT signaling (Figure S4A,B, Supporting Information). Consistent with numerous reports of persistent STAT3 pathway activation in CRC, we verified significant STAT3 activation in CRC cells relative to that in FHC cells (Figure S4C, Supporting Information). Furthermore, western blot assays showed that SNORA28 knockdown suppressed the level of STAT3 phosphorylation at the 705 tyrosine residue (Tyr705) and vice versa (Figure 3B,C). Immunofluorescence staining revealed that SNORA28 overexpression enhances the nuclear translocation of STAT3 (Figure 3D). STAT3 is primarily phosphorylated by JAKs (especially JAK1 and JAK2). However, this was not detected due to the low endogenous expression levels of p‐JAK1 and p‐JAK2. We used RNA interference (siRNA) to verify whether JAKs were involved in SNORA28‐induced STAT3 activation. As shown in Figure 3E, JAK1 siRNA and not JAK2 siRNA blocked SNORA28‐activated STAT3 phosphorylation.

To further investigate whether the JAK1/STAT3 pathway is required for SNORA28‐driven CRC cell proliferation and radioresistance, Niclosamide, which is a small‐molecule STAT3 inhibitor,^[^ ^25^ ^]^ was used to treat CRC cells that stably overexpressed SNORA28. The data showed that the p‐STAT3 (Tyr705) levels were significantly suppressed (Figure 3F), and the promoting effects of SNORA28 overexpression on the CRC cell proliferation, colony formation, and radioresistance were reversed by Niclosamide (Figure 3G–J). This indicated that the JAK1/STAT3 pathway mediates the biological effects of SNORA28 on CRC cells. Subsequently, we detected the effects of SNORA28 on the STAT3 signaling after irradiation. LV‐SNORA28+IR group exhibited higher p‐STAT3 levels compared with those in the LV‐NC+IR group (Figure S4D, Supporting Information). Thus, the continued activation of STAT3 signaling in SNORA28‐overexpressing irradiated CRC cells may facilitate acquired radioresistance. Previous studies highlighted Survivin as a downstream target gene of the STAT3 pathway.^[^ ^26^ ^]^ Our results demonstrated that SNORA28 positively regulates the expression of Survivin. Moreover, rescue experiments have indicated that inhibiting the STAT3 pathway significantly attenuates SNORA28‐induced Survivin expression (Figure 3K–M).

### 2.4 SNORA28 Activates the JAK1/STAT3 Pathway via LIFR

**Figure 4:**
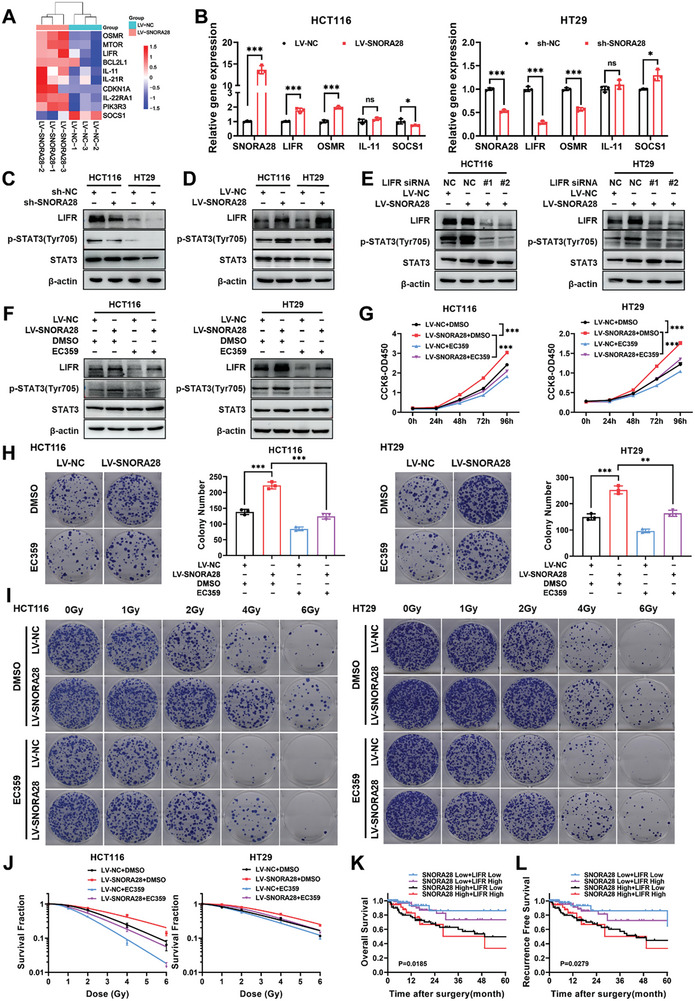
LIFR is required for SNORA28‐activated JAK1/STAT3 pathway. A) Heat map analysis of differentially expressed genes in the cytokine–cytokine receptor interaction pathway. B) qRT‐PCR verification of differentially expressed genes. C–D) Western blotting analysis of the LIFR, p‐STAT3, and STAT3 protein levels after SNORA28 knockdown (C) and overexpression (D). E–F) Western blotting analysis of the indicated proteins in SNORA28 overexpressing CRC cells treated with LIFR siRNA (E) for 48 h or 5 µM EC359 for 24 h (F). G–H) CCK8 (G) and colony formation (H) analyses of the proliferation (*n* = 5) and survival ability (*n* = 3) in SNORA28 overexpressing CRC cells treated with 5 µM EC359 for 24 h. I–J) Colony formation assays (I) and survival curves (J) analyses of radiosensitivity in SNORA28 overexpressing CRC cells treated with 5 µM EC359 for 24 h (*n* = 3). K–L) Kaplan–Meier analyses of OS (K) and RFS (L) for patients with CRC (*n* = 476) under the combination of SNORA28 and LIFR. Data are represented as the means ± SD. ^*^ *p* < 0.05; ^**^ *p* < 0.01; ^***^ *p* < 0.001; ns, no significance.

KEGG signaling pathway enrichment analysis revealed that the top‐ranked enriched term was the cytokine–cytokine receptor pathway, which could activate the JAK/STAT signaling. To further confirm the detailed molecular mechanism underlying JAK1/STAT3 pathway activation by SNORA28, differentially expressed genes in the cytokine–cytokine receptor pathway were heat‐mapped and preliminarily verified (**Figure** **4**A). SNORA28 overexpression significantly increased LIFR and OSMR mRNA levels while significantly suppressing SOCS1 expression in HCT116 cells. Conversely, SNORA28 knockdown had the opposite effects in HT29 cells (Figure 4B). However, the downregulation of SOCS1 protein expression following SNORA28 knocked down or overexpression indicated that SOCS1 is not a key mediator in the SNORA28‐regulated JAK1/STAT3 pathway (Figure S5A,B, Supporting Information). SNORA28 knockdown cells exhibited significantly lower protein levels of LIFR and OSMR than those in control cells, while SNORA28‐overexpressing cells showed significantly higher LIFR and OSMR levels than in control cells (Figure 4C,D; Figure S5A,B, Supporting Information). In addition, siRNAs against LIFR and OSMR blocked SNORA28‐induced p‐STAT3 expression (Figure 4E; Figure S5C, Supporting Information), suggesting that LIFR and OSMR are potential downstream targets of SNORA28 that aid in the activation of the JAK1/STAT3 signaling pathway.

To clarify whether LIFR was necessary for SNORA28 to induce the oncogenic phenotype in CRC cells, we performed rescue assays in SNORA28‐overexpressing CRC cells using EC359, which is a specific LIFR inhibitor. We found that EC359 effectively attenuated the LIFR and p‐STAT3 protein levels upregulated by SNORA28 (Figure 4F). Additionally, SNORA28‐mediated cell proliferation, survival, and radioresistance were reversed by EC359 (Figure 4G–J). Although SNORA28 positively regulated the expression of OSMR, which functions as an oncogene that is associated with poor prognosis in CRC (Figure S5D, Supporting Information), OSMR knockdown did not affect the promoting effect of SNORA28 on CRC cell proliferation and colony formation (Figure S5E,F, Supporting Information). These results showed that LIFR rather than OSMR is the downstream target of SNORA28 that mediates proliferation and radioresistance in CRC cells.

Furthermore, we explored the significance of SNORA28 as a clinical prognostic indicator when used in combination with LIFR. Compared with patients with CRC in the low‐expression SNORA28/LIFR group, both the OS and RFS of those with CRC in the high‐expression SNORA28/LIFR group were shorter (Figure 4K,L). This indicates that the SNORA28/LIFR pathway promotes CRC progression.

### 2.5 Histone Acetylation Drives SNORA28 to Regulate LIFR Expression

**Figure 5:**
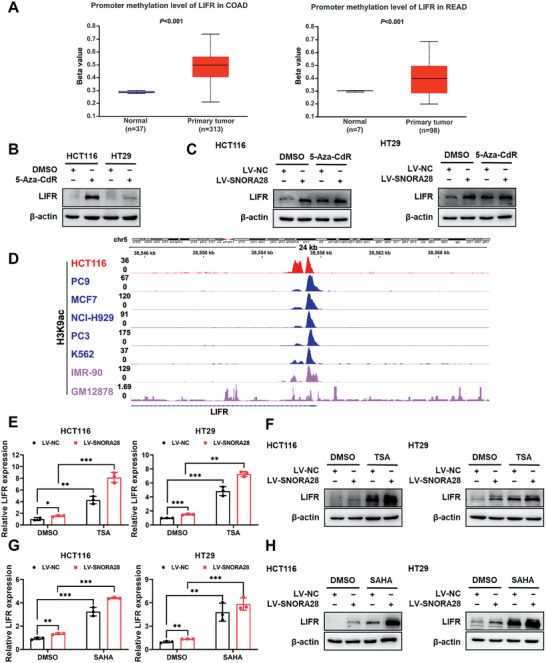
Histone acetylation drives SNORA28‐induced LIFR expression. A) UALCAN database analysis of LIFR promoter methylation levels in COAD and READ. B) Western blotting assays showing LIFR protein levels in CRC cells following treatment with 10 µM 5‐Aza‐CdR for 72 h. C) Western blotting analysis of LIFR levels in CRC cells with stable SNORA28 overexpression after 10 µM 5‐Aza‐CdR treatment for 72 h. D) ChIP‐seq data from the ENCODE project showing H3K9ac signal at the LIFR locus of the genome browser (hg38) in HCT116 cells and other cell types (PC9: non‐small cell lung cancer, MCF7: breast cancer, NCI‐H929: myeloma, PC3: prostatic cancer, K562: chronic myelogenous leukemia, IMR‐90: Human embryonic fibroblasts, GM12878: Human B lymphocyte). E–H) mRNA and protein levels of LIFR in CRC cells with stable SNORA28 overexpression after 500 nM Trichostatin A (TSA) treatment for 12 h (E, F) and 5 µM Vorinostat (SAHA) treatment for 12 h (G, H). Data are represented as the means ± SD. *n* ≥ 3. ^**^ *p* < 0.01, ^***^ *p* < 0.001.

Next, we explored the molecular mechanisms underlying SNORA28‐induced upregulation of LIFR transcription. Previous reports demonstrated that the CpG islands in the LIFR promoter region of CRC cells are methylated.^[^ ^27^ ^]^ Based on this, we used the UALCAN database for further analysis and found LIFR hypermethylation in colon adenocarcinoma (COAD) and rectum adenocarcinoma (READ) tissues in comparison with normal tissues (**Figure** **5**A). DNA methylation often leads to gene downregulation or silencing. To ascertain the correlation between SNORA28‐upregulated LIFR expression and DNA methylation, HCT116 and HT29 cells were treated with 5‐Aza‐2'‐deoxycytidine (5‐Aza‐CdR), which inhibits DNA methyltransferase. We found that LIFR expression was successfully recovered by 5‐Aza‐CdR (Figure 5B). These data indicate that LIFR expression is upregulated after demethylation, confirming previous reports of LIFR promoter methylation in CRC. Further, 5‐Aza‐CdR increased LIFR protein levels in the LV‐NC group but not in the LV‐SNORA28 group (Figure 5C), suggesting that DNA methylation did not contribute to SNORA28‐mediated upregulation of LIFR expression.

Apart from DNA methylation, histone acetylation is another important epigenetic modification, which activates the transcription of target genes. Previous studies have identified histone H3 lysine 9 acetylation (H3K9ac) in the LIFR promoter region.^[^ ^28^ ^]^ Therefore, we analyzed the H3K9ac CHIP‐seq data pertaining to HCT116 cells from the Encyclopedia of DNA Elements (ENCODE) database to identify genomic sites enriched for H3K9ac. These data indicated significant H3K9ac enrichment within the LIFR promoter region. In addition, we mined H3K9ac CHIP‐seq data for other cell lines that exhibited both common and uncommon peaks with those of HCT116 cells (Figure 5D). Based on the observed data, we speculated that SNORA28 may mediate LIFR upregulation by regulating histone acetylation in the LIFR promoter region. Thus, we analyzed the effects of the two histone deacetylase inhibitors, Trichostatin A (TSA) and Vorinostat (SAHA), on LIFR expression in CRC cells. Both TSA and SAHA significantly increased the upregulation effect of SNORA28 on LIFR mRNA and protein levels (Figure 5E–H), suggesting that histone acetylation rather than DNA methylation drives the SNORA28‐mediated regulation of LIFR expression.

### 2.6 SNORA28 Recruits BRD4 to Enhance Histone Acetylation in the LIFR Promoter Region and Promote LIFR Transcription

**Figure 6:**
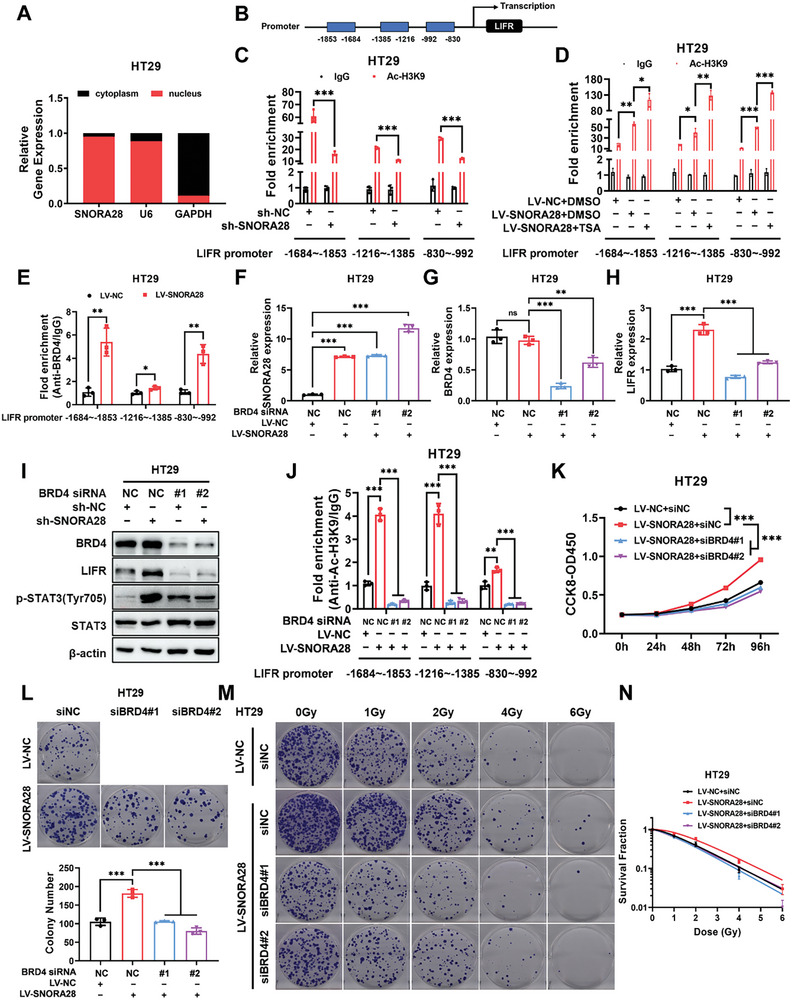
SNORA28 recruits BRD4 to increase H3K9 acetylation in the LIFR promoter. A) The subcellular localization of SNORA28 in HT29 cells was analyzed using qRT‐PCR. B) Primers were designed to target three different sites in the LIFR promoter region. C–D) CUT&RUN‐qPCR assay for assessment of H3K9ac levels in the LIFR promoter after SNORA28 knockdown (C) and overexpression (D). E) CUT&RUN‐qPCR assay for detecting BRD4 levels in LIFR promoter after SNORA28 overexpression. F–H) qRT‐PCR analysis of the mRNA levels of SNORA28 (F), BRD4 (G), and LIFR (H) in HT29 cells treated with BRD4 siRNA for 48 h following SNORA28 overexpression. I) Western blotting analysis of the indicated proteins in cells treated as described in F–H. J) CUT&RUN‐qPCR analysis of the enrichment levels of H3K9ac in LIFR promoter in cells treated as described in F–H. K–L) CCK8 (K) and colony formation (L) assays of the proliferation ability in cells treated as described in F–H. M–N) Colony formation assays (M) and survival curves (N) analyses reflect the radiosensitivity in cells treated as described in F–H. Data are represented as the means ± SD. n ≥ 3. ^*^ *p* < 0.05; ^**^ *p* < 0.01; ^***^ *p* < 0.001; ns, no significance.

To explore the precise mechanisms underlying the regulation of the LIFR/JAK1/STAT3 pathway activity by SNORA28 in CRC, we examined the subcellular localization of SNORA28. Nuclear and cytoplasmic fractionation assays revealed that SNORA28 was predominantly localized within the nucleus (**Figure** **6**A). These data suggest that SNORA28 may affect LIFR transcription by regulating histone acetylation. Therefore, we designed three pairs of primers specific to different sites of the LIFR promoter region (Figure 6B) and detected the levels of H3K9 acetylation in the LIFR promoter region after the knockdown and overexpression of SNORA28, with TSA treatment used as the positive control. CUT&RUN‐qPCR indicated that SNORA28 knockdown reduced H3K9ac enrichment in the LIFR promoter region (Figure 6C). In contrast, SNORA28 overexpression increased H3K9 acetylation level, which became significantly higher after TSA treatment (Figure 6D). These data confirm that SNORA28 enhances histone acetylation in the LIFR promoter.

Bromodomain‐containing protein 4 (BRD4) recruitment enhances H3K9ac enrichment at the LIFR promoter region and activates its transcription.^[^ ^29^ ^]^ Therefore, we speculate that SNORA28 may regulate histone acetylation at the LIFR promoter region via BRD4. First, CUT&RUN‐qPCR results confirmed that SNORA28 overexpression recruits BRD4 to the LIFR promoter (Figure 6E). Second, the siRNA against BRD4 effectively suppressed SNORA28‐upregulated LIFR mRNA and protein levels (Figure 6F–I) and significantly attenuated the SNORA28‐mediated increase in H3K9ac levels at the LIFR promoter region (Figure 6J). Lastly, BRD4 knockdown was confirmed to reverse SNORA28‐induced cell proliferation, colony formation, and radioresistance in HT29 cells (Figure 6K–N). Similar results were observed in HCT116 cells (Figure S6, Supporting Information). These results show that SNORA28 recruits BRD4 to enhance H3K9 acetylation at the LIFR promoter region and activate LIFR transcription.

### 2.7 SNORA28 Overexpression Enhances CRC Cell Growth and Radioresistance In Vivo

Having established that SNORA28 activates the LIFR/JAK1/STAT3 pathway to enhance CRC cell proliferation and radioresistance in vitro, we established mouse xenograft models of HT29/LV‐SNORA28 and HT29/LV‐NC to analyze its effect on CRC growth and radiosensitivity in vivo and elucidate its modulatory effect on the LIFR/JAK1/STAT3 pathway.

**Figure 7:**
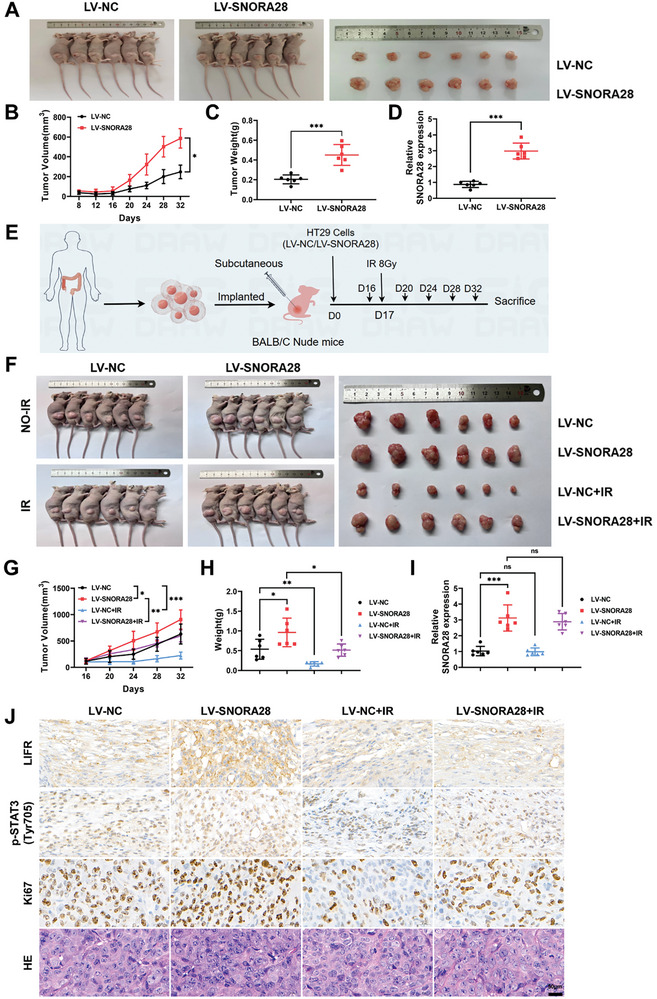
SNORA28 overexpression enhances xenograft tumor growth and radioresistance. A) Representative images of SNORA28‐overexpressing HT29 xenograft tumors. B, C) Growth curves (B) and weight (C) of the transplanted tumors. D) SNORA28 expression levels in xenograft tumors. E) Schematic representation of radiation exposure in nude mice with transplanted tumors. F) Representative images of SNORA28‐overexpressing HT29 xenograft tumors after irradiation. G–H) Growth curves (G) and weight (H) of the transplanted tumors after irradiation. I) SNORA28 expression levels in xenograft tumors after irradiation. J) Hematoxylin and eosin (H&E) and immunohistochemistry (IHC) analysis of xenograft tumors after irradiation (100  ×). Bars, 50 µm. *n* = 6. ^*^ *p* < 0.05; ^**^ *p* < 0.01; ^***^ *p* < 0.001.

First, we found that the SNORA28‐overexpression group mice exhibited tumors that were larger and heavier than those of the control group mice (**Figure** **7**A–D). Second, administering a single dose of irradiation (8 Gy) to the irradiation group comprised of randomly grouped nude mice whose xenograft tumor volumes had reached approximately 100 mm^3^ produced significant inhibition of tumor growth. However, the degree of inhibition in the LV‐SNORA28+IR group was much lower than that in the LV‐NC+IR group, indicating that SNORA28 overexpression enhances in vivo radioresistance in CRC (Figure 7E–I). Finally, immunohistochemistry (IHC) analysis showed that the intensity of positive staining for Ki67, LIFR, and p‐STAT3 in the SNORA28 overexpression group was significantly stronger than that in the control group. Meanwhile, irradiation significantly suppressed the expression of these proteins. Nevertheless, the expression of these proteins in the LV‐SNORA28+IR group was still stronger than that in the LV‐NC+IR group (Figure 7J). Overall, these results indicate that SNORA28 overexpression enhances the growth and radioresistance of xenograft tumors and activates the LIFR/JAK1/STAT3 pathway in vivo, which is consistent with our in vitro results.

## 3 Discussion

**Figure 8:**
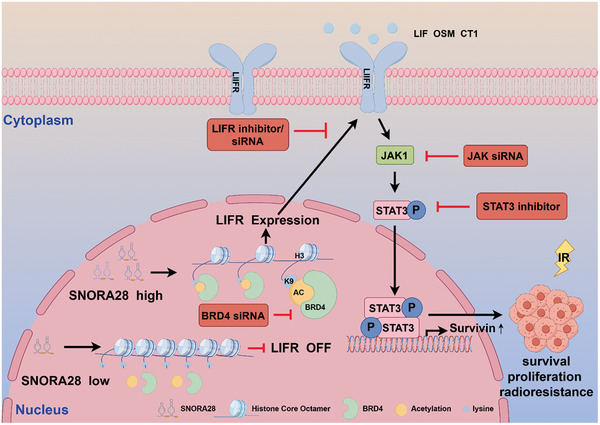
A schematic model of SNORA28‐based regulatory mechanism in CRC.

Approximately 50% of cancer patients require radiotherapy, which is an essential part of tumor treatment.^[^ ^30^ ^]^ However, CRC cells tend to show low radiosensitivity,^[^ ^31^ ^]^ which limits the efficacy of radiotherapy in patients with CRC. Therefore, identifying radiosensitizing therapeutic targets is of great clinical significance as it would improve the outcomes of radiotherapy‐based treatment regimens in these patients. In the present study, we found that SNORA28 regulates the growth of CRC cells and enhances their radioresistance, implying that snoRNAs directly regulate the radiosensitivity of tumor cells. Moreover, SNORA28 may act as a molecular decoy by recruiting BRD4 to increase H3K9 acetylation in the LIFR promoter, which in turn promotes LIFR transcription and activates the JAK1/STAT3 pathway. Thus, snoRNAs play a role in chromatin remodeling by mediating histone modifications in the promoter region of target genes (**Figure** **8**).

Initially, snoRNAs were generally regarded as housekeeping genes with limited effects on cellular homeostasis.^[^ ^32^ ^]^ However, in‐depth studies of their role in tumorigenesis and development have gradually revealed relevant roles in CRC. For example, SNORD1C was shown to maintain stemness and chemoresistance by activating the Wnt signaling pathway in CRC.^[^ ^10^ ^]^ SNORD33 is downregulated in CRC and functions as a tumor suppressor.^[^ ^33^ ^]^ While the role of snoRNAs in regulating tumor cell growth and chemotherapy resistance is now well‐established,^[^ ^10^, ^14^ ^]^ their influence on radiosensitivity has rarely been studied. Our data show that SNORA28 is highly expressed in CRC tissues, and its expression positively correlates with poor prognosis of patients with CRC. Moreover, SNORA28 promoted CRC growth and radioresistance, as confirmed through in vitro and in vivo experiments. Thus, our results shed light on the expression pattern and biological function of SNORA28 in CRC, in addition to providing insights into how snoRNAs may regulate tumor cell radiosensitivity, which highlights their potential as radiosensitizing targets.

The underlying reason for SNORA28 upregulation in CRC currently remains unclear. In mammals, snoRNAs are transcribed from host gene introns, lack independent promoters, and thus depend on host gene transcription and splicing for expression. SNORA28 is located in the intron region of the EIF5 gene on chromosome 14q32.32 and is 126 nt in length, belonging to the class of H/ACA box snoRNAs. SNORA28 may be expressed via transcription and splicing of the host gene EIF5. Transcription factors may be binding to the EIF5 promoter region thus regulating the expression of both EIF5 and SNORA28. Moreover, we cannot rule out that their expression is subject to epigenetic regulation, such as promoter hypomethylation or promoter histone acetylation. In addition, SNORA28 may also bind to the core proteins of H/ACA small nucleolar ribonucleoproteins (snoRNPs), which would prevent its degradation by exonucleases, thereby stabilizing its levels.

SnoRNAs were shown to be closely related to multiple signal pathways, including the Wnt/β‐catenin,^[^ ^34^ ^]^ PI3K/AKT,^[^ ^13b^ ^]^ and p53 pathway.^[^ ^35^ ^]^ However, the relationship between snoRNAs and the STAT3 pathway is rather unclear. In this study, we found that SNORA28 positively regulates p‐STAT3 (Tyr705) expression, and Niclosamide (a STAT3 inhibitor) significantly blocks the stimulatory effect of SNORA28 on CRC cell proliferation and radioresistance. Thus, the SNORA28/STAT3 axis, which is critical for CRC progression, could represent a therapeutic target in patients with CRC, especially in radioresistant cases.

To determine the specific process underlying SNORA28‐mediated regulation of STAT3 phosphorylation, we analyzed RNA‐seq data following SNORA28 overexpression. We identified LIFR, OSMR, and SOCS1, three factors enriched in the cytokine–cytokine receptor pathway, as potential candidates that may be involved in the SNORA28‐induced activation of p‐STAT3 (Tyr705). Specific inhibitor and RNA interference experiments revealed that LIFR, rather than OSMR or SOCS1, is responsible for SNORA28‐regulated p‐STAT3 expression, which in turn promotes CRC cell proliferation and radioresistance. LIFR is a classical cytokine receptor that is not self‐activated and requires specific binding to ligands (e.g., LIF, CT1, and OSM). Due to frequent DNA methylation of the CpG islands in the LIFR promoter region in various types of tumors,^[^ ^36^ ^]^ including CRC,^[^ ^27a^ ^]^ its role in solid tumors is controversial and requires further study. Therefore, we analyzed the prognostic value of combining SNORA28 expression with that of LIFR. Patients with high SNORA28/LIFR expression had shorter overall survival (OS) and recurrence‐free survival (RFS) compared to those without high expression, suggesting that LIFR functions as an oncogene in CRC and that the SNORA28/LIFR pathway promotes CRC progression. Similarly, Wu et al.^[^ ^37^ ^]^ discovered that LIFR is an oncogene that promotes the growth, migration, and angiogenesis of CRC, which is consistent with our findings.

Histone acetylation is an important epigenetic modification involved in transcription, chromatin remodeling, and DNA repair.^[^ ^38^ ^]^ snoRNAs primarily guide the post‐transcriptional modification and maturation of target RNAs, such as rRNAs, snRNAs, and mRNA.^[^ ^39^ ^]^ snoRNAs may function as miRNA sponges^[^ ^40^ ^]^ or piRNAs,^[^ ^41^ ^]^ and directly interact with proteins to regulate target gene expression.^[^ ^42^ ^]^ Nevertheless, the role of snoRNAs in histone acetylation is yet to be reported. Herein, we showed that SNORA28 recruits BRD4, increases H3K9 acetylation in the LIFR promoter region, and “unlocks” LIFR expression through the acetylation‐mediated opening of the chromatin structure granting transcription factors access to the LIFR promoter. This also suggests a role for SNORA28 in chromatin remodeling. Taken together, our findings reveal that snoRNAs regulate target gene transcription by altering the chromatin structure. Therefore, further analysis of the similarities and differences between the structure of SNORA28 and other snoRNAs may help clarify the influence of histone acetylation on snoRNA‐mediated transcriptional activation of target genes.

Bromine domain and outer end (BET) proteins, histone acetyltransferases (HATs), and histone deacetylases (HDACs) dynamically regulate histone acetylation by recognizing, adding, or removing acetyl groups from lysine residues.^[^ ^43^ ^]^ Bromodomain‐containing protein 4 (BRD4) is a member of the BET family. As a key acetylated lysine reader, BRD4 preferentially acetylates histone H3 at Lysine (K) residues 9, 14, and 27 as well as histone H4 at K5, K8, K12, and K16, thus regulating transcription initiation and elongation.^[^ ^44^ ^]^ Meanwhile, BRD4 displays histone acetyltransferase activity,^[^ ^45^ ^]^ which mediates the expression of multiple genes such as MMP2,^[^ ^46^ ^]^ CXCL8,^[^ ^47^ ^]^ and IDO1.^[^ ^48^ ^]^ In the present study, we found that BRD4 regulates LIFR transcription. siRNA against BRD4 effectively suppressed the SNORA28‐mediated upregulation of LIFR mRNA and protein levels, significantly reduced H3K9ac levels within the LIFR promoter, and successfully reversed SNORA28‐induced CRC cell proliferation and radioresistance. These findings shed light on the molecular mechanism of SNORA28‐mediated CRC radioresistance. However, the detailed interactions underlying this mechanism and the binding sites between SNORA28 and BRD4 require further investigation. Several recent studies have shown that targeting BRD4 inhibits CRC progression and chemoresistance.^[^ ^49^ ^]^ Our study complements the current understanding of its role in the efficacy of CRC radiotherapy and guides novel strategies for its application in the treatment of CRC.

Taken together, this study is the first to demonstrate that snoRNAs regulate the radiosensitivity of CRC cells and are involved in chromatin remodeling by mediating histone modifications in the promoter region of target genes. Furthermore, SNORA28 activates the LIFR/JAK1/STAT3 pathway to promote CRC progression and radioresistance, which highlights its potential as a therapeutic target in CRC.

## 4 Experimental Section

### Bioinformatics Analysis

The expression of SNORA28 in CRC patient samples and its association with survival was evaluated using the data from the TCGA database (https://tcgadata.nci.nih.gov/tcga). The correlation between various clinicopathological parameters and SNORA28 expression was analyzed using the same data. SNORA28 expression in samples from patients with other types of cancer was analyzed using the SNORic database (http://bioinfo.life.hust.edu.cn/SNORic). LIFR methylation status in COAD and READ tissues was analyzed using the UALCAN database (http://ualcan.path.uab.edu/). The signaling pathways related to SNORA28 were analyzed using the Database for Annotation, Visualization, and Integrated Discovery (DAVID, https://david.ncifcrf.gov/home.jsp). The overall survival of CRC patients based on OSMR expression levels was analyzed using the GEPIA database (http://gepia2.cancer‐pku.cn/).

### Patients and Samples

In total, 57 pairs of CRC and adjacent normal tissue specimens were collected from patients with stage I–IV CRC who underwent tumor resection at the Department of Colorectal Surgery, Cancer Hospital of China Medical University. Three pairs of clinical samples were used for nrStar Human snoRNA PCR chip analysis. The snoRNA PCR chip data reported in this study were stored in NCBI GEO with accession number: GSE267087. The clinical information of the three participants is shown in Table S3 (Supporting Information). The use of these specimens was approved by the Ethics Committee on Human Investigation of the Liaoning Cancer Hospital, and informed consent was obtained from each patient. The clinical details of the 57 patients with CRC are shown in Table S4 (Supporting Information).

### Reagents

STAT3 inhibitor Niclosamide (MedChemExpress # BAY2353), LIFR inhibitor EC359 (MedChemExpress # HY‐120142), DNA methyltransferase inhibitor 5‐Aza‐CdR (Selleck # NSC127716), histone deacetylase inhibitors TSA (MedChemExpress # HY‐15144) and SAHA (MedChemExpress # HY‐10221).

### Cell Culture

The human normal colon epithelial cell line (FHC) was obtained from the American Type Culture Collection (ATCC, Manassas, Virginia, USA), human small intestinal epithelial cells (HIEC‐6) were preserved by the laboratory, and human CRC cell lines (HCT116, HT29, and SW620) were obtained from GeneChem (Shanghai, China). FHC cells were maintained in DMEM: F‐12 medium (ATCC, USA), supplemented with10% fetal bovine serum (FBS, Gibco, USA), 20  ng mL^−1^ human recombinant EGF (Thermo Fisher PHG0311), 100 ng mL^−1^ hydrocortisone, 0.005 mg mL^−1^ transferrin, 10 ng m^−1^L cholera toxin, and 10 mM HEPES (at a final concentration of 25 mM). HIEC6 cells were cultured in opti‐MEM medium (Gibco, USA), supplemented with 10% fetal bovine serum (FBS, Gibco, USA). HT29 cells were cultured in high‐glucose Dulbecco's Modified Eagle Medium (DMEM) (Gibco, USA), supplemented with 10% FBS (ExCell Bio, Shanghai, China). HCT116 and SW620 cells were cultured in RPMI‐1640 medium (Gibco, USA), supplemented with 10% FBS (ExCell Bio, China). All cells were maintained in a medium supplemented with 1% penicillin/streptomycin solution and cultured in an incubator containing 5% CO2 at 37 °C. According to the requirements of the experiment, the cells were treated with the STAT3 inhibitor (Niclosamide, 2.5 µM for 12 h), the LIFR inhibitor (EC359, 5 µM for 24 h), the DNA methyltransferase inhibitor (5‐Aza‐CdR, 10 µM for 72 h), or two histone deacetylase inhibitors (TSA, 500 nM for 12 h; SAHA, 5 µM for 12 h).

### Cell Transfection

Cells were transfected with shRNA targeting SNORA28 (sh‐SNORA28) using Lipofectamine (Invitrogen, 11668‐019). Lentiviruses (GV235 vector) overexpressing SNORA28 and the negative control lentivirus were infected using HiTransG P. Cells stably overexpressing SNORA28 were selected with 2 µg mL^−1^ puromycin for 72 h. Both the shRNA and lentiviruses were synthesized and purchased from GeneChem. All RNAi oligonucleotides were purchased from Shanghai GenePharma. Cells were transfected with siRNA duplexes using Lipofectamine (Invitrogen, 11668‐019) by following the manufacturer's instructions. Oligonucleotide sequences were used as follows:

Sh‐SNORA28 (5′–3′): AGCAACACTCTGTGGCAGAT;
Sh‐NC (5′–3′): TTCTCCGAACGTGTCACGT.
LV‐SNORA28 (5′–3′): (AgeI restriction endonuclease site) ACCGGTAAGCAACACTCTGTGGCAGATGATCAAAACTGTCTGACACAATTTG
AGCTTGCTATAGCAAGAAAGTCTAACCTATTCCGGTGTTCTCTCTCCCATGA
GACAAGCCGTTATATAGACTTAAACAGTGAATTC (EcoRI restriction endonuclease site);
LV‐NC (5′–3′): TTCTCCGAACGTGTCACGT.

Details of all the siRNA sequences are provided in Table S4 (Supporting Information).

### qRT‐PCR

Total RNA was extracted from tissues or cells using TRIzol reagent (Invitrogen, USA), following the manufacturer's protocol. Reverse transcription of RNA into cDNA was performed using PrimeScript RT Reagent (Takara, Japan). qRT‐PCR was performed using the CFX Connect Real‐Time PCR Detection System (Bio‐Rad, USA) with SYBR Green mix (Toyobo, Japan) and specific primers. U6 and GAPDH were used as reference genes for snoRNA and mRNA expression, respectively. Primer sequences are shown in Table S5 (Supporting Information).

### Cell Proliferation Assay

Cell proliferation was assessed using the CCK‐8 assay kit (Dojindo, Kumamoto, Japan), according to the manufacturer's instructions. Cells were seeded at a density of 2 × 10^3^ cells/well in 96‐well plates with five replicates. After incubation for 0, 24, 48, 72, and 96 h, 10% CCK8 reagent was added, and the cells were incubated for 2 h. The absorbance was measured at 450 nm using a microplate reader (Bio‐Rad, 170–6750).

### Colony Formation Assay

The survival ability of cells was analyzed via colony formation assay: 600 cells were seeded in 6‐well plates and cultured for 12 days. The medium was changed every 4 days. All cell colonies were fixed with methanol for 30 min and stained with Giemsa for 1 h at 37 °C. The radiosensitivity of the cells was analyzed via colony formation assay: 1200 cells were seeded in 6‐well plates, cultured for 24 h, and then irradiated with 0,1, 2, 4, and 6 Gy of ^60^Co γ‐ray with a 66.44 cGy min^−1^ dose rate. The culture medium was changed every 4 days. After 12 days of culture, all the cell colonies were fixed with methanol for 30 min, and stained with Giemsa for 1 h at 37 °C. The number of colonies and the sensitizing enhancement ratio (SER) were calculated, and the survival curve after irradiation was fitted according to the single‐hit multitarget model: SF = 1‐(1‐e^−kD^) ^n^. Each experiment was performed in triplicate.

### Cell Apoptosis Assay

Different treatment groups were stained using the Annexin V‐FITC Apoptosis Detection Kit (Dojindo, Kumamoto, Japan). The treated cells were harvested via trypsinization and washed in pre‐cooled PBS. The cells were then stained with Annexin V‐FITC and propyl iodide (PI) mixture at 25 °C for 15 min, away from light. The percentage of apoptotic cells was measured using flow cytometry (ACEA Bio, San Diego, CA, USA). Each experiment was performed in triplicates.

### Immunofluorescence Assay

Coverslips were placed in a 6‐well plate, and 2×10^5^ cells of different groups (LV‐NC, LV‐SNORA28) were cultured on the coverslip. After culturing for 48 h, the cells were fixed with 4% paraformaldehyde, the cell's membrane was permeated with 0.4% Triton X‐100 ‐PBS buffer, and the cells were blocked with 5% BSA‐0.3% Triton X‐100 ‐PBS buffer for 1 h. Subsequently, the STAT3 antibody (Cell Signaling Technology; # 9139, 1:100) was added and incubated at 4 °C overnight. After three washes with PBS, sheep anti‐mouse IgG (Mouse Alexa; #594, 1:200) was incubated at room temperature for 2 h, and 20 µL of fluorescent sealing agent containing DAPI was added to the coverslip. Finally, the results were observed and imaged using a fluorescence microscope (Nikon, Tokyo, Japan).

### RNA‐Sequencing

HCT116 cells were infected with LV‐NC and LV‐SNORA28 lentivirus for 72 h, and samples were collected. Total RNA was isolated using TRIzol reagent (Invitrogen, Carlsbad, CA, USA) following the manufacturer's procedure. The RNA amount and purity of each sample were quantified using NanoDrop ND‐1000 (NanoDrop, Wilmington, DE, USA). RNA was purified, fragmented, and reverse‐transcribed by Dynabeads Oligo (dT) (Thermo Fisher, CA, USA), Magnesium RNA Fragmentation Module (NEB, cat. e6150, USA), and SuperScript II Reverse Transcriptase (Invitrogen, cat. 1 896 649, USA). Subsequently, cDNA libraries were constructed. Finally, 2 × 150 bp paired‐end sequencing (PE150) was performed on an Illumina Novaseq 6000 (LC‐Bio Technology CO., Ltd., Hangzhou, China) according to the vendor's recommended protocol. The GEO accession number for the RNA‐seq data was GSE267012.

### Animal Experiment

HT29 cells (1 × 10^7^ cells) infected with LV‐NC or LV‐SNORA28 were subcutaneously injected into the 4‐week‐old BALB/c nude mice (*n* = 6). The size of the tumor was measured every 4 days, and the tumor volume was calculated based on the formula (length×width^2^)/2. When the xenograft tumor volume reached approximately 100 mm^3^, nude mice were randomly allocated into groups (LV‐NC, LV‐SNORA28, LV‐NC+IR, LV‐SNORA28+IR) and received local irradiation with 8 Gy (68.99 R min^−1^ dose rate) in the irradiation group. After 32 days, subcutaneous xenografts from different groups were collected, imaged, and weighed. H&E staining and IHC for Ki67(Abcam; # ab15580, 1:4000), p‐STAT3(ABclonal; # AP0705, 1:100), and LIFR (Santa Cruz Biotechnology; # sc‐515337, 1:400) were performed. All animal experiments were performed in compliance with the regulations of the Ethics Committee of the Academy of Military Medical Sciences (IACUC‐DWZX‐2022‐833; Beijing, China).

### Western Blotting

Cells were lysed in RIPA buffer containing phosphatase and protease inhibitors. The protein concentration was determined using the BCA assay kit (Beyotime # P0010). Equal amounts of total protein from different groups were separated via 8–12% SDS‐PAGE and then electroblotted onto an NC membrane. After blocking with 5% fat‐free milk powder, the membrane was incubated with specific primary antibodies overnight at 4°C. These included antibodies against JAK1 (Cell Signaling Technology; # 29 261, 1:1000), JAK2 (Cell Signaling Technology; # 3230, 1:1000), STAT3 (Cell Signaling Technology; # 9139, 1:1000), p‐STAT3 (Cell Signaling Technology; # 9145, 1:500), SOCS1 (Abcam; # ab280886, 1:1000), OSMR (Santa Cruz Biotechnology; # sc‐271695, 1:100), LIFR (Santa Cruz Biotechnology; # sc‐515337, 1:100), BRD4 (Cell Signaling Technology; # 13 440, 1:1000), β‐actin (Proteintech; # 60008‐1‐Ig, 1:5000), mouse IgG (HRP‐conjugated) (SeraCare; # 5220‐0341, 1:5000), rabbit IgG (HRP‐conjugated) (SeraCare; # 5220‐0336,1:5000). The membrane was washed with TBS‐Tween 20 (TBS‐T) three times and then incubated with mouse IgG (SeraCare; # 5220‐0341,1:5000) or rabbit IgG (SeraCare; # 5220‐0336, 1:5000) secondary antibodies for 1.5 h, followed by visualization of the bands with an ECL kit (Thermo Fisher # 34 580).

### CUR&RUN‐qPCR

CUT&RUN‐qPCR was performed using a Hyperactive pG‐MNase CUT&RUN Assay Kit (Vazyme; # HD101‐01). Different groups of cells were incubated with ConA Beads Pro at 37 °C for 10 min to permeate the cell membrane. Cells were then incubated with anti‐Acetyl‐Histone H3 (Lys9) (Cell Signaling Technology; # 9649, 1:50) and anti‐BRD4 (Cell Signaling Technology; # 13 440, 1:50) antibodies. The isotype control was incubated with an equal amount of rabbit IgG (Beyotime; # A7016). Incubation was performed overnight at 4 °C. Subsequently, pG‐MNase Enzyme was diluted with MNase Dilution Buffer at the ratio of 1:100, and the diluted solution was fully mixed with the cell‐magnetic bead complex, followed by incubation for 1 h at 4 °C with shaking. CaCl_2_ was then added, and the sample was placed on ice 90 min for digestion, then incubated for 30 min at 37 °C. Chromatin enrichment products were collected, and DNA was extracted. Finally, qPCR was performed to detect the target genes. The specific primers used are shown in Table S5 (Supporting Information).

### Statistical Analyses

Data for each group were normalized using the mean value of three replicates from the control group. All experiments were performed in triplicate, and all data were analyzed as the mean ± standard deviation (SD), and differences between two groups were compared with a two‐tailed unpaired Student's t‐test. The relation between SNORA28 expression and clinicopathological parameters in patients with CRC was evaluated using the Chi‐square (χ^2^) test. Survival analyses were performed using the Kaplan–Meier method and log‐rank test. A Cox proportional hazards regression model was used to identify independent prognostic factors related to survival. GraphPad Prism (version 9.0) software and SPSS (version 26.0) were used for statistical analysis. *p*‐values < 0.05 were considered statistically significant; ^***^ *p* < 0.001, ^**^ *p *< 0.01, ^*^ *p* < 0.05. ns, no statistical significance.

## Conflict of Interest

The authors declare no conflict of interest.

## Author Contributions

X. L. and H. Z. contributed equally to this work. X.L. and H.Z. performed most of the experiments, analyzed data, and wrote the first draft of the manuscript. Y.F., D.C., and R.L. performed sections of the experiments. Q.W., Y.L., L.S., and Y.G. involved in data collection and analysis. Q.Z., Z.Q., and Z.W. contributed conception and design of the experiments. Z.W. edited the manuscript. All authors approved the final manuscript.

## Supporting information

Supporting Information

## Data Availability

The data that support the findings of this study are openly available in GEO at https://www.ncbi.nlm.nih.gov/geo/query/acc.cgi?acc=GSE267087, reference number 267087.
