# Supplementary material for: SNORA28 Promotes Proliferation and Radioresistance in Colorectal Cancer Cells through the STAT3 Pathway by Increasing H3K9 Acetylation in the LIFR Promoter
Source: Adv Sci (Weinh). 2024 Jun 26;11(32):2405332. doi: 10.1002/advs.202405332 (PMC11347989; doi:10.1002/advs.202405332)
Supplement: Supplementary file 1 — Supporting Information [file ADVS-11-2405332-s001.docx]

Supporting Information

**SNORA28 Promotes Proliferation and Radioresistance in Colorectal Cancer Cells through the STAT3 Pathway by Increasing H3K9 Acetylation in the LIFR Promoter**

Xin Liu, Hong Zhang, Ying Fan, Dan Cai, Ridan Lei, Qi Wang, Yaqiong Li, Liping Shen, Yongqing Gu, Qingtong Zhang,* Zhenhua Qi,* Zhidong Wang*


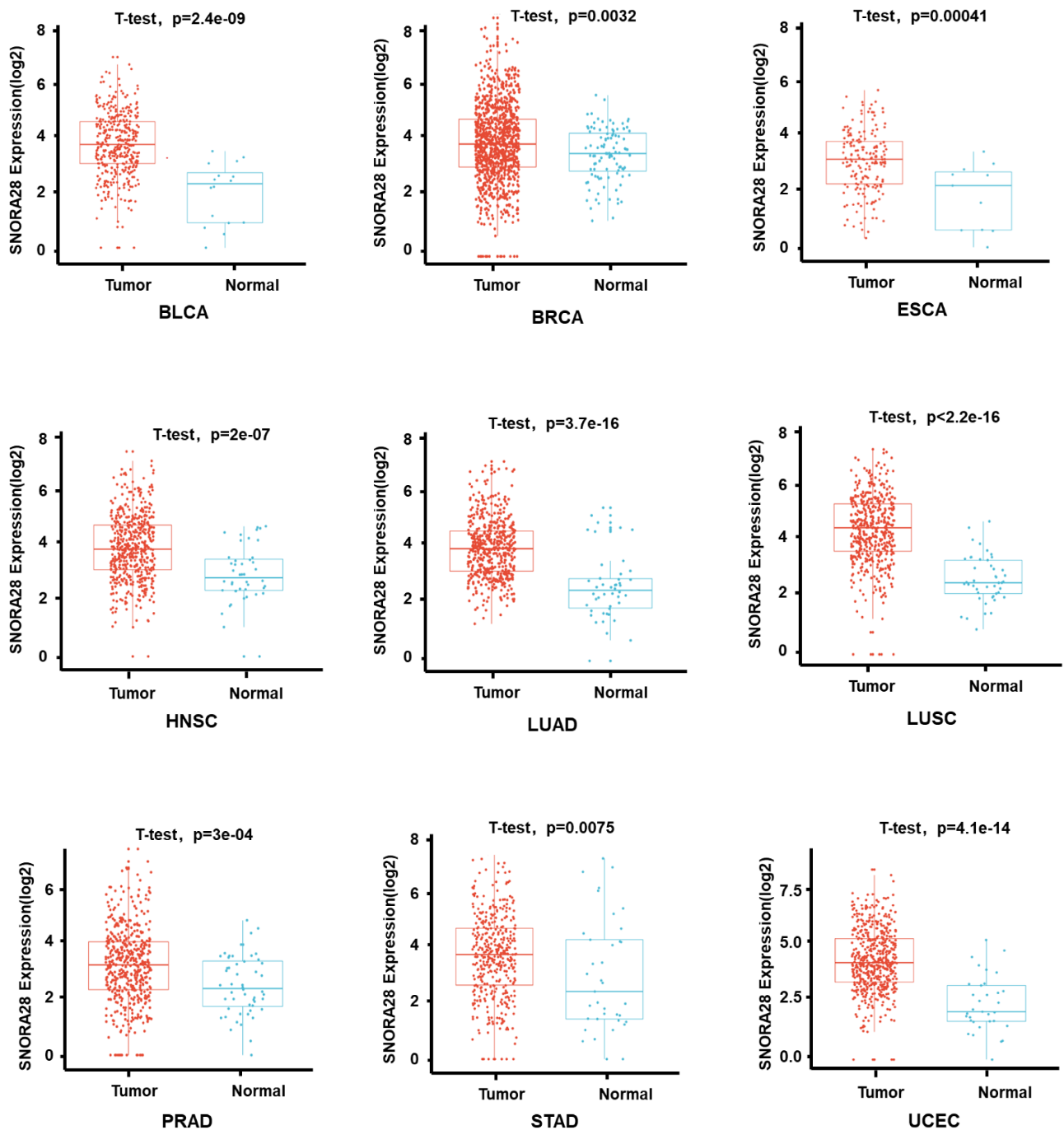


**Figure S1.** SNORA28 was highly expressed in multiple types of tumors.


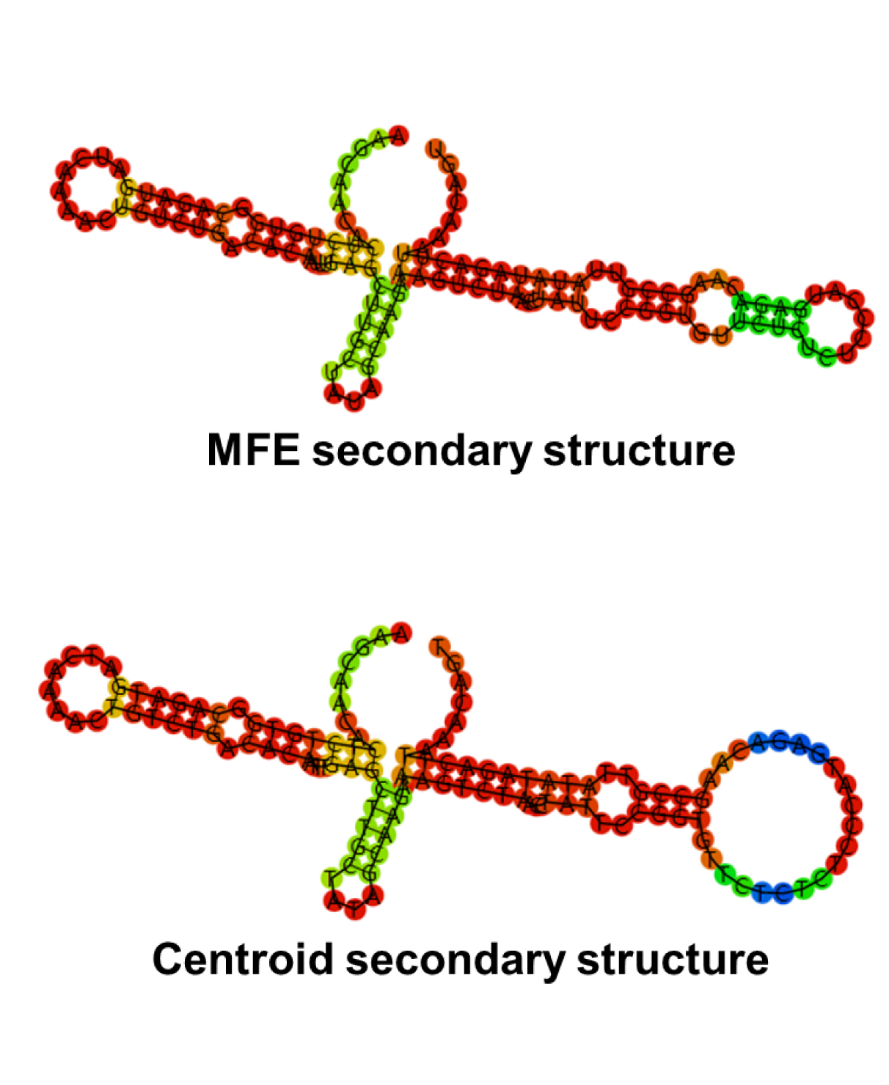


**Figure S2.** The predicted secondary structure of SNORA28.


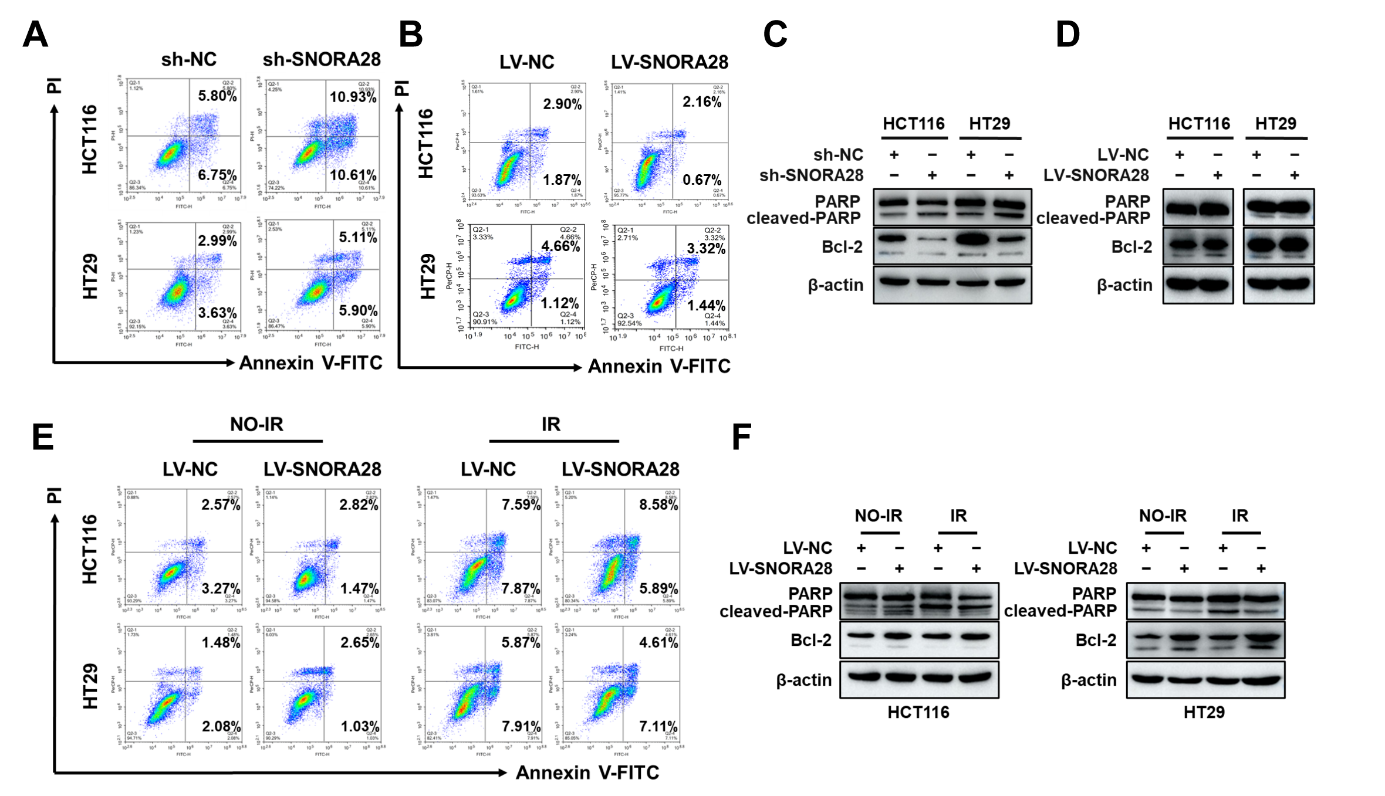


**Figure S3**. The effect of SNORA28 on cell apoptosis in both non-irradiated and irradiated conditions. A–B) Flow cytometric analyses of CRC cell apoptotic rates after SNORA28 knockdown (A) or overexpression (B). C–D) Western blotting analysis of PARP and Bcl-2 protein levels in CRC cells after SNORA28 knockdown (C) or overexpression (D). E) Flow cytometry analysis of the apoptotic rates post-irradiated (8Gy) SNORA28-overexpressing CRC cells for 48 h. F) Western blotting analysis the levels of PARP and Bcl-2 protein after post-irradiated (8Gy) SNORA28-overexpressing CRC cells for 48 h.


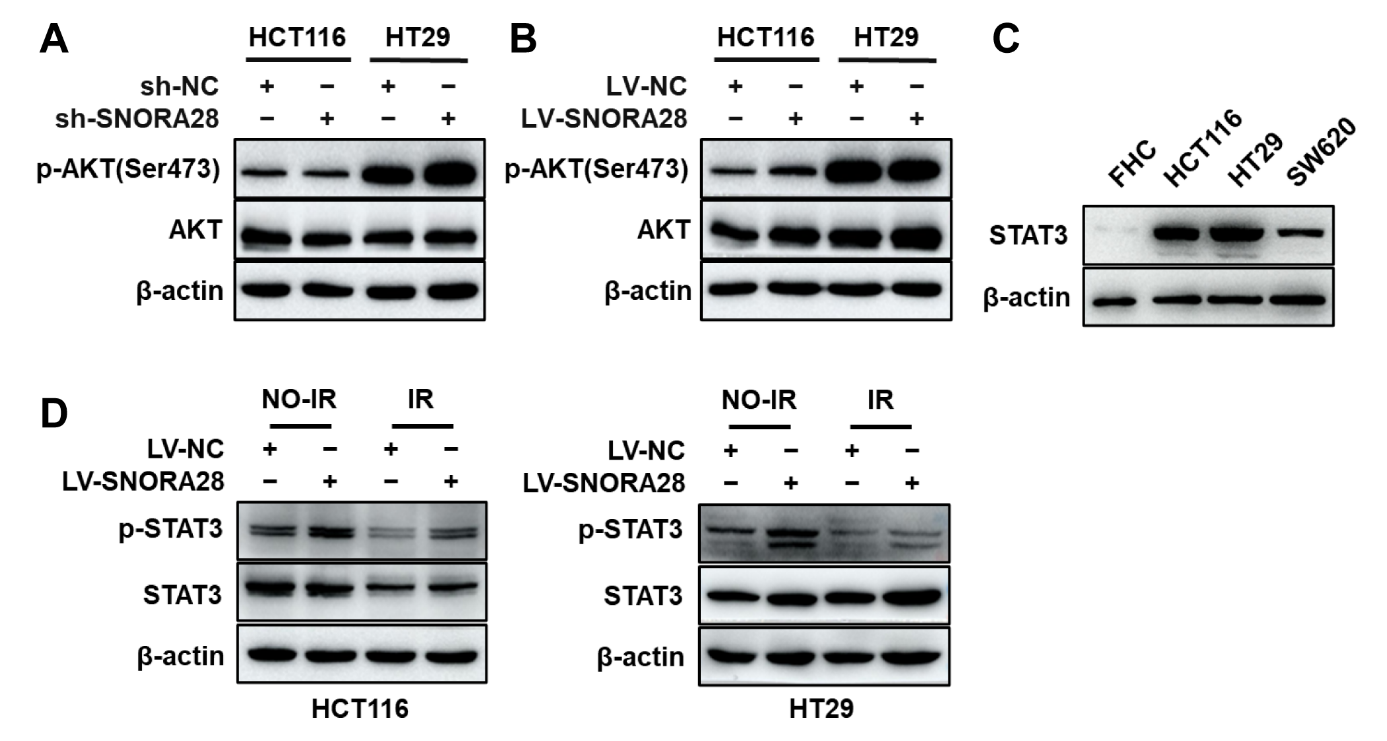


**Figure S4.** SNORA28-mediated biological effects independent of the PI3K/AKT pathway. A–B) Western blot analysis of the PI3K/AKT pathway proteins in CRC cells after SNORA28 knockdown (A) and overexpression (B). C) Protein expression of STAT3 in the three CRC cell lines and the normal FHC cells. D) Western blot analysis of the STAT3 pathway proteins expression levels post-irradiation (8Gy) for 48 h. n ≥ 3. ns, no significance.


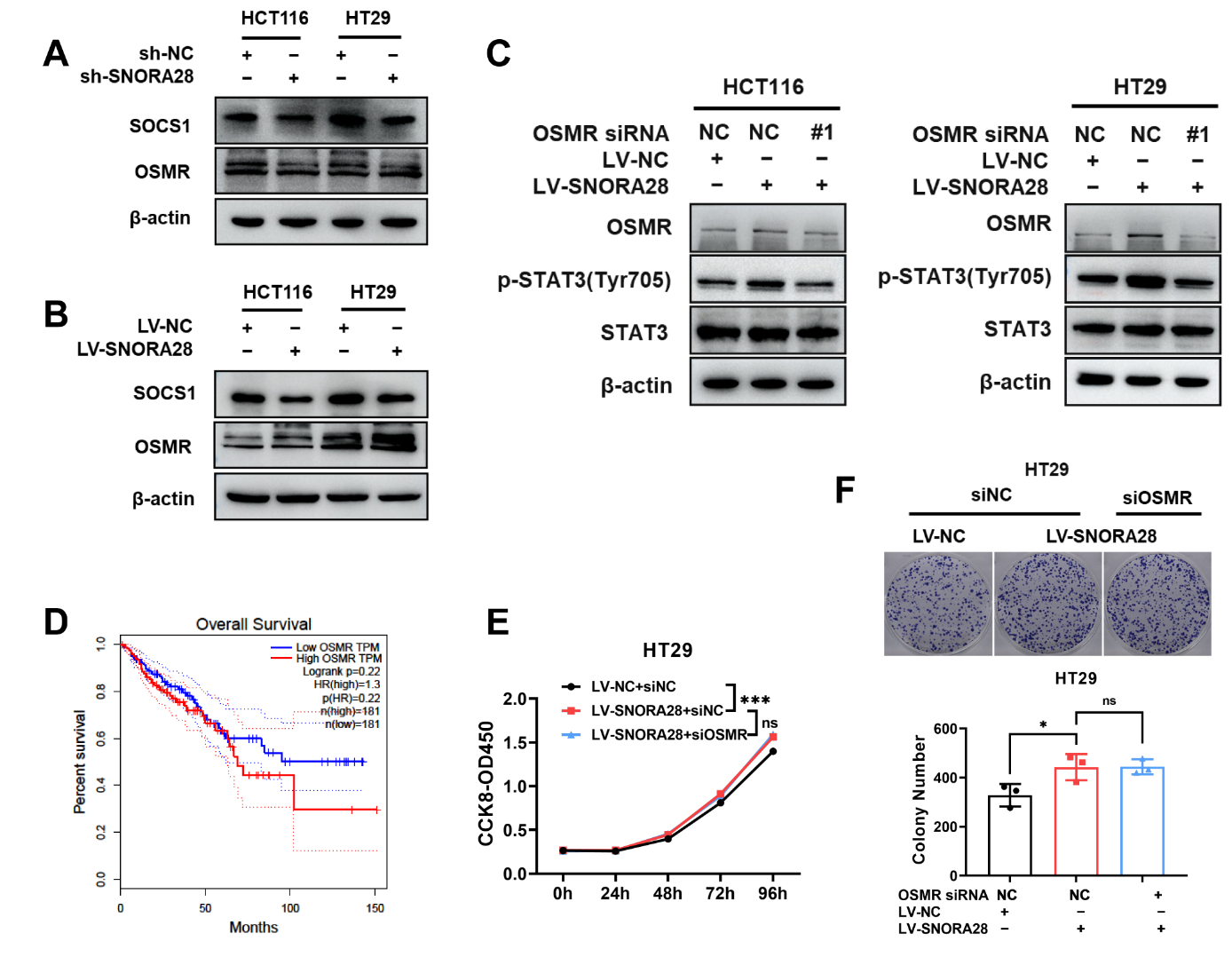


**Figure S5.** SNORA28-mediated biological effects were independent of SOCS1 and OSMR. A–B) Western blot analysis of SOCS1 and OSMR protein expression levels in CRC cells after SNORA28 knockdown (A) and overexpression (B). C) Western blot analysis of the OSMR and STAT3 pathway proteins in cells treated with OSMR siRNA under SNORA28 overexpression. D) GEPIA database analysis of overall survival for CRC patients based on OSMR expression levels. E–F) CCK8 (E) and colony formation (F) analysis of the proliferation ability in HT29 cells treated with OSMR siRNA in the presence of SNORA28 overexpression. Data are represented as the means ± SD. n ≥ 3. * *P* < 0.05; *** *P* < 0.001; ns, no significance.


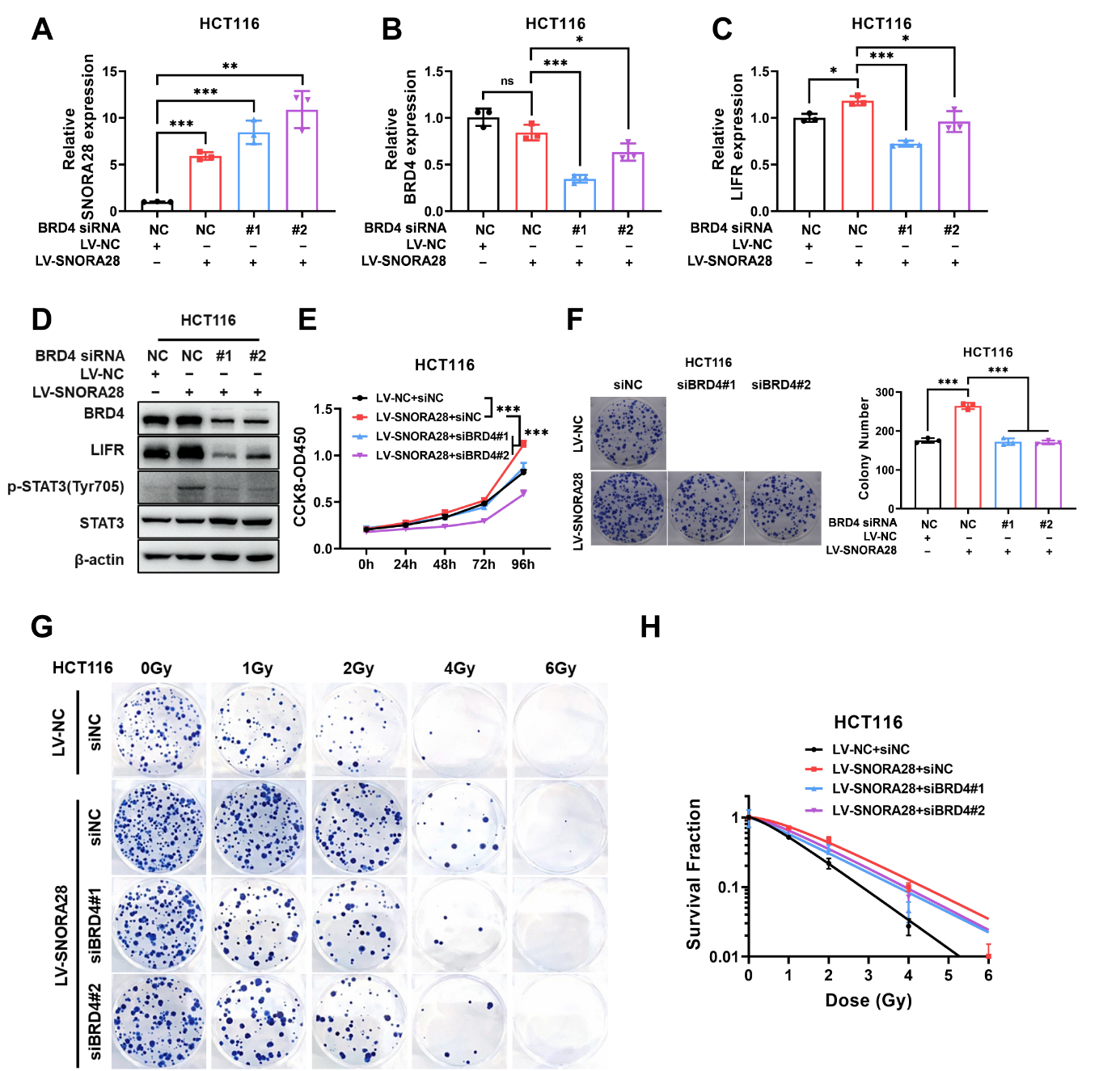


**Figure S6.** BRD4 knockdown reversed SNORA28-induced CRC cell proliferation and radioresistance in HCT116 cells. A–C) qRT-PCR analyses of the mRNA expression levels of SNORA28 (A), BRD4 (B), and LIFR (C) in HCT116 cells treated with BRD4 siRNA in the presence of SNORA28 overexpression. D) Western blot analysis of the indicated proteins in cells treated as in described A–C. E–F) CCK8 (E) and colony formation (F) assays of the proliferation ability in cells treated as in described A–C. G–H) Colony formation assays (G) and survival curves (H) reflecting the radiosensitivity of cells treated as described in A–C. Data are represented as the means ± SD. n ≥ 3. * *P* < 0.05; ** *P* < 0.01; *** *P* < 0.001; ns, no significance.

**Table S1.** Heatmap data of snoRNA microarray in this study.

| Gene name | Tumor1 | Tumor2 | Tumor3 | Normal1 | Normal2 | Normal3 | regulation |
| --- | --- | --- | --- | --- | --- | --- | --- |
| U56/SNORD56 | 0.07611 | 0.01251 | 0.0063 | 0.00034 | 0.00161 | 0.00166 | UP |
| U19/SNORA74A | 0.00732 | 0.00302 | 0.00911 | 4E-05 | 0.00013 | 0.00124 | UP |
| U83/SNORD117 | 0.00591 | 0.00069 | 0.00151 | 4.1E-05 | 5.3E-05 | 0.00038 | UP |
| U12snRNA | 0.28745 | 0.2791 | 0.28871 | 0.00257 | 0.01127 | 0.07294 | UP |
| U102/SNORD102 | 0.28537 | 0.11416 | 0.16447 | 0.0029 | 0.01371 | 0.05726 | UP |
| U46/SNORD46 | 0.00416 | 0.00144 | 0.00111 | 4.3E-05 | 0.0003 | 0.00031 | UP |
| U45A/SNORD45A | 0.01015 | 0.00662 | 0.00644 | 0.00011 | 0.00031 | 0.00107 | UP |
| U45C/SNORD45C | 0.00323 | 0.00239 | 0.00207 | 4E-05 | 0.00036 | 0.00045 | UP |
| U80/SNORD80 | 0.14952 | 0.09193 | 0.09374 | 0.00273 | 0.01935 | 0.02889 | UP |
| U58B/SNORD58B | 0.00492 | 0.00115 | 0.00112 | 9.2E-05 | 0.00029 | 0.0004 | UP |
| U97 | 0.00214 | 0.00121 | 0.00183 | 4E-05 | 0.0003 | 0.00038 | UP |
| U28/SNORD28 | 0.00497 | 0.00104 | 0.00149 | 0.0001 | 0.00019 | 0.00042 | UP |
| U54/SNORD54 | 0.00814 | 0.00114 | 0.00046 | 0.00017 | 0.00043 | 0.00015 | UP |
| U58C/SNORD58C | 0.00188 | 0.00048 | 0.00089 | 4E-05 | 0.00013 | 0.00014 | UP |
| U76/SNORD76 | 0.00949 | 0.01412 | 0.01465 | 0.00021 | 0.00259 | 0.00394 | UP |
| U16/SNORD16 | 0.00176 | 0.00093 | 0.00086 | 4E-05 | 0.0001 | 0.00019 | UP |
| HBII-99B/SNORD12B | 0.0192 | 0.0105 | 0.00907 | 0.00045 | 0.00126 | 0.0027 | UP |
| U108/SNORA12 | 0.00622 | 0.00229 | 0.00154 | 0.00015 | 0.00074 | 0.0007 | UP |
| HBI-115/SNORA47 | 0.01267 | 0.00333 | 0.00908 | 0.0003 | 0.00088 | 0.00271 | UP |
| U32A/SNORD32A | 0.0213 | 0.00765 | 0.00929 | 0.00053 | 0.00274 | 0.00409 | UP |
| mgh28S-2409/SNORD5 | 0.00143 | 0.00086 | 0.00094 | 4E-05 | 0.00032 | 0.00023 | UP |
| U15B/SNORD15B | 0.00264 | 0.00125 | 0.00133 | 7.7E-05 | 0.00029 | 0.00037 | UP |
| SNORD121B | 0.00162 | 0.0002 | 0.00038 | 4.7E-05 | 6.6E-05 | 4.9E-05 | UP |
| U96a/SNORD96A | 0.00136 | 0.00028 | 0.00054 | 4E-05 | 8.9E-05 | 0.00021 | UP |
| U71a/SNORA71A | 0.00151 | 0.00035 | 0.00062 | 4.5E-05 | 7.9E-05 | 0.00019 | UP |
| U41/SNORD41 | 0.01953 | 0.0048 | 0.00519 | 0.00061 | 0.00137 | 0.00183 | UP |
| ACA53/SNORA53 | 0.00277 | 0.00096 | 0.00167 | 8.8E-05 | 3.9E-05 | 0.00047 | UP |
| mgU2-19/30/SCARNA9 | 0.00123 | 0.00052 | 0.00061 | 4E-05 | 3.3E-05 | 0.00017 | UP |
| U90/SCARNA7 | 0.00251 | 0.00149 | 0.00244 | 8.6E-05 | 0.00014 | 0.00045 | UP |
| U14A/SNORD14A | 0.01627 | 0.00346 | 0.00503 | 0.00057 | 0.0004 | 0.00116 | UP |
| mgU12-22/U4-8 | 0.00924 | 0.00319 | 0.00093 | 0.00036 | 0.00095 | 0.00036 | UP |
| U60/SNORD60 | 0.00629 | 0.00325 | 0.00342 | 0.00025 | 0.00117 | 0.00132 | UP |
| HTR | 0.00127 | 0.0019 | 0.0026 | 5.1E-05 | 0.00013 | 0.00097 | UP |
| HBII-420/SNORD99 | 0.00711 | 0.01937 | 0.00624 | 0.00029 | 0.00253 | 0.00164 | UP |
| SNORD126 | 0.00212 | 0.00049 | 0.00079 | 9.2E-05 | 0.00017 | 0.00018 | UP |
| ACA5C/SNORA5C | 0.00089 | 0.00026 | 0.00013 | 4E-05 | 9.4E-05 | 3.3E-05 | UP |
| U47/SNORD47 | 0.01454 | 0.00941 | 0.00707 | 0.00072 | 0.00157 | 0.00205 | UP |
| ACA43/SNORA43 | 0.00405 | 0.0044 | 0.00251 | 0.00022 | 0.0006 | 0.00083 | UP |
| HBII-135/SNORD65 | 0.00698 | 0.003 | 0.00467 | 0.00038 | 0.0006 | 0.00104 | UP |
| U81/SNORD81 | 0.0032 | 0.00331 | 0.00242 | 0.00019 | 0.00101 | 0.00073 | UP |
| ACA33/SNORA33 | 0.00068 | 0.00112 | 0.0006 | 4E-05 | 0.00024 | 8.8E-05 | UP |
| U35B/SNORD35B | 0.00067 | 0.0005 | 0.00061 | 4E-05 | 0.00014 | 0.00023 | UP |
| U33/SNORD33 | 0.06964 | 0.03255 | 0.03102 | 0.00425 | 0.00852 | 0.01262 | UP |
| nop56p | 0.00358 | 0.0017 | 0.00097 | 0.00022 | 0.0002 | 0.00035 | UP |
| HBII-95/SNORD11 | 0.00064 | 0.00033 | 0.00023 | 4E-05 | 0.00011 | 0.00011 | UP |
| ACA28/SNORA28 | 0.00062 | 0.00029 | 9.1E-05 | 4E-05 | 3.3E-05 | 4E-05 | UP |
| U27/SNORD27 | 0.02345 | 0.00365 | 0.00524 | 0.00158 | 0.00159 | 0.00193 | UP |
| ACA18/SNORA18 | 0.00712 | 0.00179 | 0.0027 | 0.0005 | 0.0004 | 0.00083 | UP |
| mgU6-53B/SNORD9 | 0.00056 | 0.00012 | 0.00013 | 4E-05 | 3.3E-05 | 3.8E-05 | UP |
| HBII-95B/SNORD11B | 0.0005 | 0.00027 | 0.00011 | 4E-05 | 6.8E-05 | 2E-05 | UP |
| nop1p | 0.00134 | 0.00361 | 0.00194 | 0.00011 | 0.00051 | 0.00066 | UP |
| U22/SNORD22 | 0.00159 | 0.00079 | 0.00154 | 0.00013 | 0.00021 | 0.00042 | UP |
| ACA22/SNORA22 | 0.05047 | 0.00691 | 0.01148 | 0.00423 | 0.00279 | 0.00528 | UP |
| U106/SNORD12C | 0.00076 | 0.00103 | 0.00082 | 6.6E-05 | 0.00029 | 0.00027 | UP |
| U77/SNORD77 | 0.00044 | 0.00089 | 0.00088 | 4E-05 | 0.00028 | 0.00015 | UP |
| U101/SNORD101 | 0.00126 | 0.0005 | 0.00033 | 0.00011 | 0.00022 | 8.9E-05 | UP |
| ACA21/SNORA21 | 0.0053 | 0.00055 | 0.00264 | 0.00051 | 0.00022 | 0.00091 | UP |
| u67/SNORA67 | 0.003 | 0.00109 | 0.00201 | 0.00029 | 0.00037 | 0.00059 | UP |
| U4snRNA | 0.15391 | 0.0521 | 0.10407 | 0.01512 | 0.00698 | 0.006 | UP |
| ACA56/SNORA56 | 0.02116 | 0.0031 | 0.00383 | 0.0021 | 0.00144 | 0.00155 | UP |
| U36A/SNORD36A | 0.01259 | 0.0076 | 0.00733 | 0.00127 | 0.00092 | 0.00167 | UP |
| NOP58P | 0.00035 | 0.00051 | 0.00029 | 4E-05 | 3.3E-05 | 8.8E-05 | UP |
| HBI-6/SNORA26 | 0.00182 | 0.00083 | 0.00053 | 0.00023 | 0.00028 | 0.00026 | UP |
| ACA35/SCARNA1 | 0.00041 | 0.0008 | 0.00035 | 5.3E-05 | 6.7E-05 | 0.00014 | UP |
| U31/SNORD31 | 0.00031 | 0.00019 | 0.00017 | 4E-05 | 3.5E-05 | 7.2E-05 | UP |
| U48/SNORD48 | 0.12449 | 0.01309 | 0.01865 | 0.01677 | 0.00529 | 0.0075 | UP |
| U87/SCARNA5 | 0.0066 | 0.00179 | 0.00106 | 0.00139 | 0.00042 | 0.00051 | UP |
| U35A/SNORD35A | 0.00427 | 0.00134 | 0.00107 | 0.00096 | 0.00046 | 0.0004 | UP |
| ACA24/SNORA24 | 0.00318 | 0.00204 | 0.0018 | 0.00074 | 0.00084 | 0.00047 | UP |
| U36C/SNORD36C | 0.02348 | 0.00491 | 0.00463 | 0.00562 | 0.00224 | 0.0023 | UP |
| SNORA36C | 0.00016 | 9.9E-05 | 0.00011 | 4E-05 | 3.3E-05 | 4.4E-05 | UP |
| U44/SNORD44 | 0.01134 | 0.00663 | 0.00557 | 0.00319 | 0.00254 | 0.00195 | UP |
| u65/SNORA65 | 0.00493 | 0.00159 | 0.00149 | 0.00157 | 0.00057 | 0.00065 | UP |
| ACA44/SNORA44 | 0.00011 | 0.00039 | 0.00015 | 4E-05 | 3.8E-05 | 1.9E-05 | UP |
| HBII-99/SNORD12 | 0.00041 | 0.00079 | 0.00054 | 0.00015 | 0.00017 | 0.00013 | UP |
| ACA32/SNORA32 | 0.00143 | 0.00071 | 0.00066 | 0.00053 | 0.0002 | 0.00028 | UP |
| U49B/SNORD49B | 0.00118 | 0.00243 | 0.00134 | 0.00048 | 0.00042 | 0.00047 | UP |
| U17b/SNORA73B | 0.00437 | 0.00513 | 0.00456 | 0.00208 | 0.0023 | 0.0014 | UP |
| ACA64/SNORA78 | 2E-05 | 1.6E-05 | 1.4E-05 | 4E-05 | 3.3E-05 | 7.3E-05 | DOWN |
| SNORD75L2 | 5E-05 | 2.2E-06 | 2E-06 | 0.0001 | 0.00021 | 8.9E-06 | DOWN |
| 14q(II-1) (1,8,27)/SNORD114-1 | 0.00079 | 0.00013 | 6E-05 | 0.00162 | 0.00115 | 0.00035 | DOWN |
| U92/SCARNA8 | 0.00269 | 0.00014 | 0.00014 | 0.00579 | 0.00133 | 0.00086 | DOWN |
| U43/SNORD43 | 0.00171 | 0.00036 | 0.00024 | 0.00384 | 0.00383 | 0.00062 | DOWN |
| U63/SNORD63 | 0.05899 | 0.00736 | 0.01305 | 0.14891 | 0.1276 | 0.03278 | DOWN |
| SNORD62BL1 | 1.4E-05 | 1.3E-05 | 3.4E-06 | 4E-05 | 0.00019 | 7.9E-06 | DOWN |
| 14q(II-15) (3,5,21,23,25,26)/SNORD114-15 | 1.5E-05 | 4E-06 | 2.5E-06 | 4.6E-05 | 0.00013 | 1.4E-05 | DOWN |
| mgU2-25/61 | 0.10719 | 0.01021 | 0.00535 | 0.33966 | 0.17287 | 0.05896 | DOWN |
| ACA66 | 0.00442 | 0.0002 | 0.00027 | 0.01408 | 0.00822 | 0.00266 | DOWN |
| 14q(II-11)/SNORD114-11 | 3E-05 | 1.6E-06 | 2.4E-06 | 9.9E-05 | 3.3E-05 | 7.9E-06 | DOWN |
| HBII-52-12/SNORD115-12 | 2.6E-05 | 6.3E-06 | 1.9E-05 | 9E-05 | 0.00011 | 4E-05 | DOWN |
| 14q(II-7)/SNORD114-7 | 1.1E-05 | 3.1E-06 | 8.3E-07 | 4E-05 | 4.1E-05 | 7.9E-06 | DOWN |
| 14q(I-1)/SNORD113-1 | 1.1E-05 | 5.1E-06 | 3.6E-06 | 4E-05 | 0.00016 | 7.9E-06 | DOWN |
| HBII-419/SNORD98 | 0.00975 | 0.00084 | 0.00031 | 0.03907 | 0.01415 | 0.00551 | DOWN |
| SNOED39 | 9.7E-06 | 1.6E-06 | 8.3E-07 | 4E-05 | 0.00035 | 7.9E-06 | DOWN |
| SNORD118L9 | 9.6E-06 | 8.6E-07 | 1.7E-06 | 4E-05 | 0.0002 | 1.2E-05 | DOWN |
| SNORA20L4 | 9E-06 | 7E-07 | 8.3E-07 | 4E-05 | 3.3E-05 | 7.9E-06 | DOWN |
| 14q(II-2)/SNORD114-2 | 9E-06 | 5.9E-06 | 6.2E-06 | 4E-05 | 3.3E-05 | 1.3E-05 | DOWN |
| ACA16/SNORA16 | 0.00085 | 0.00084 | 0.00031 | 0.00408 | 0.00192 | 0.00068 | DOWN |
| SNORA25L14 | 8.3E-06 | 2.3E-06 | 4.9E-06 | 4E-05 | 3.3E-05 | 2.4E-05 | DOWN |
| 14q(I-5)/SNORD113-5 | 1.1E-05 | 1.6E-06 | 8.3E-07 | 5.8E-05 | 3.6E-05 | 7.9E-06 | DOWN |
| ACA61/SNORA61 | 0.52724 | 0.10789 | 0.02266 | 2.85374 | 1.67118 | 0.53443 | DOWN |
| 14q(I-4)/SNORD113-4 | 1.1E-05 | 4.9E-06 | 1.8E-06 | 6.2E-05 | 7.5E-05 | 7.9E-06 | DOWN |
| SNORA38B/ACA38B | 0.00185 | 6.1E-05 | 9.7E-05 | 0.01099 | 0.00111 | 0.00083 | DOWN |
| HBII-52-36/SNORD115-36 | 0.00014 | 1.5E-05 | 4.4E-05 | 0.00083 | 0.00352 | 0.00013 | DOWN |
| SNORD118L14 | 2.3E-05 | 3E-06 | 3.6E-06 | 0.00015 | 0.00043 | 9.1E-06 | DOWN |
| 14q(II-20) (21,22,28)/SNORD114-20 | 2.6E-05 | 1.3E-05 | 7E-06 | 0.00017 | 0.00025 | 4.9E-05 | DOWN |
| HBII-438A/B(SNORD109A/B) | 7E-06 | 2.1E-06 | 3.4E-06 | 4.6E-05 | 0.00014 | 7.9E-06 | DOWN |
| 14q (0)/SNORD112 | 1.3E-05 | 6.4E-06 | 8.3E-07 | 8.6E-05 | 0.00015 | 7.9E-06 | DOWN |
| 14q(II-13)/SNORD114-13 | 5.6E-06 | 1.7E-06 | 1.6E-06 | 4E-05 | 6.3E-05 | 7.9E-06 | DOWN |
| U68/SNORA68 | 0.0596 | 0.00747 | 0.00419 | 0.44907 | 0.07551 | 0.03732 | DOWN |
| HBI-36/SNORA35 | 9.3E-06 | 7.6E-07 | 1.2E-06 | 7.2E-05 | 6.8E-05 | 7.9E-06 | DOWN |
| ACA11/SCARNA22 | 0.0001 | 7.2E-06 | 1.5E-05 | 0.00083 | 3.3E-05 | 0.00072 | DOWN |
| 14q(II-10)/(II-18)/SNORD114-10 | 4.2E-06 | 4.9E-06 | 3.3E-06 | 4E-05 | 5.9E-05 | 7.9E-06 | DOWN |
| SNORA32L2 | 8.9E-06 | 7E-07 | 8.3E-07 | 8.7E-05 | 3.3E-05 | 7.9E-06 | DOWN |
| 14q(I-7)/SNORD113-7 | 4.1E-06 | 2.4E-06 | 1.5E-06 | 4E-05 | 0.00014 | 7.9E-06 | DOWN |
| SNORD74L4 | 1.1E-05 | 1.8E-06 | 8.3E-07 | 0.00011 | 0.00019 | 7.9E-06 | DOWN |
| SNORD45BL1 | 4.7E-06 | 7E-07 | 8.3E-07 | 4.7E-05 | 9.8E-05 | 7.9E-06 | DOWN |
| 14q(I-2)/SNORD113-2 | 4E-06 | 1.4E-06 | 1.3E-06 | 4E-05 | 3.3E-05 | 7.9E-06 | DOWN |
| 14q(I-9)/(I-3)/SNORD113-9 | 4E-06 | 1.9E-06 | 8.3E-07 | 4E-05 | 5.4E-05 | 7.9E-06 | DOWN |
| 14q(II-16)/SNORD114-16 | 4E-06 | 2E-06 | 8.3E-07 | 4E-05 | 3.3E-05 | 7.9E-06 | DOWN |
| 14q(II-31)/SNORD114-31 | 4E-06 | 7.4E-07 | 3.4E-06 | 4E-05 | 0.0001 | 1.3E-05 | DOWN |
| 14q(II-6)/II-9/SNORD114-6 | 4E-06 | 7E-07 | 8.3E-07 | 4E-05 | 4.2E-05 | 7.9E-06 | DOWN |
| AL137790.4 | 4E-06 | 2E-06 | 8.3E-07 | 4E-05 | 3.3E-05 | 1.2E-05 | DOWN |
| HBII-52-28/SNORD115-28 | 4E-06 | 1.1E-06 | 3.5E-06 | 4E-05 | 3.3E-05 | 7.9E-06 | DOWN |
| HBII-52-37/SNORD115-37 | 4E-06 | 7E-07 | 8.3E-07 | 4E-05 | 6.1E-05 | 8.4E-06 | DOWN |
| HBII-52-48/SNORD115-48 | 4E-06 | 7E-07 | 2.6E-06 | 4E-05 | 4.5E-05 | 7.9E-06 | DOWN |
| SNORA10L1 | 4E-06 | 1.9E-06 | 8.3E-07 | 4E-05 | 7.8E-05 | 7.9E-06 | DOWN |
| SNORA11C/U107D | 4E-06 | 7E-07 | 8.3E-07 | 4E-05 | 3.3E-05 | 7.9E-06 | DOWN |
| SNORA18L1 | 4E-06 | 7E-07 | 8.3E-07 | 4E-05 | 0.00014 | 9.2E-06 | DOWN |
| SNORA64L2 | 4E-06 | 1.1E-05 | 1.7E-06 | 4E-05 | 0.00012 | 7.9E-06 | DOWN |
| SNORA72L7 | 4E-06 | 7E-07 | 8.3E-07 | 4E-05 | 3.3E-05 | 7.9E-06 | DOWN |
| SNORD114-15L1 | 4E-06 | 7E-07 | 8.3E-07 | 4E-05 | 6E-05 | 7.9E-06 | DOWN |
| SNORD118L11 | 4E-06 | 7E-07 | 8.3E-07 | 4E-05 | 9E-05 | 7.9E-06 | DOWN |
| SNORD23 | 4E-06 | 1.2E-06 | 2.2E-06 | 4E-05 | 5.9E-05 | 7.9E-06 | DOWN |
| SNORD3@L19 | 4E-06 | 1.6E-06 | 8.3E-07 | 4E-05 | 0.00012 | 7.9E-06 | DOWN |
| SNORD3L3 | 4E-06 | 7E-07 | 8.3E-07 | 4E-05 | 3.3E-05 | 7.9E-06 | DOWN |
| SNORD51L1 | 4E-06 | 8.4E-06 | 8.3E-07 | 4E-05 | 3.3E-05 | 7.9E-06 | DOWN |
| SNORD68L1 | 4E-06 | 7E-07 | 8.3E-07 | 4E-05 | 9.1E-05 | 7.9E-06 | DOWN |
| SNORD74L5 | 4E-06 | 7E-07 | 8.3E-07 | 4E-05 | 4.3E-05 | 7.9E-06 | DOWN |
| U3.41-201 | 4E-06 | 1.1E-06 | 1.8E-06 | 4E-05 | 4.5E-05 | 7.9E-06 | DOWN |
| HBII-52-1/SNORD115-1 | 0.00011 | 1.8E-05 | 6.5E-05 | 0.00138 | 0.0055 | 0.00017 | DOWN |
| SNORD42BL1 | 5.8E-06 | 1.6E-06 | 4E-06 | 7.2E-05 | 0.00012 | 7.9E-06 | DOWN |
| SNORA51L11 | 1.5E-05 | 7E-07 | 1E-06 | 0.00019 | 9E-05 | 2E-05 | DOWN |
| U5snRNA | 0.0651 | 0.00687 | 0.00206 | 0.91748 | 0.48798 | 0.13751 | DOWN |
| 14q(II-19)/SNORD114-19 | 7.9E-06 | 7.5E-07 | 1.7E-06 | 0.00011 | 3.3E-05 | 7.9E-06 | DOWN |
| SNORD3@L37 | 6E-06 | 7E-07 | 1.2E-06 | 0.00012 | 0.00015 | 7.9E-06 | DOWN |
| SNORD3@L39 | 4E-06 | 8.9E-07 | 1.2E-06 | 0.00021 | 8.5E-05 | 7.9E-06 | DOWN |

**Table S2.** Data analysis of RNA-seq in this study.

| TermID | Description | Category | GeneRatio | P_value | P_adjust | GeneCount |
| --- | --- | --- | --- | --- | --- | --- |
| hsa04060 | Cytokine-cytokine receptor interaction | Environmental Information Processing | 98/1428 | 1.26E-10 | 1.96E-08 | 98 |
| hsa04151 | PI3K-Akt signaling pathway | Environmental Information Processing | 94/1428 | 0.0003603 | 0.00797814 | 94 |
| hsa04080 | Neuroactive ligand-receptor interaction | Environmental Information Processing | 93/1428 | 4.41E-08 | 4.56E-06 | 93 |
| hsa04514 | Cell adhesion molecules (CAMs) | Environmental Information Processing | 55/1428 | 1.82E-07 | 1.41E-05 | 55 |
| hsa04630 | Jak-STAT signaling pathway | Environmental Information Processing | 53/1428 | 2.81E-05 | 0.00145108 | 53 |
| hsa04640 | Hematopoietic cell lineage | Organismal Systems | 47/1428 | 6.85E-11 | 1.96E-08 | 47 |
| hsa05162 | Measles | Human Diseases | 42/1428 | 0.0010446 | 0.01541959 | 42 |
| hsa05146 | Amoebiasis | Human Diseases | 37/1428 | 1.20E-05 | 0.00074469 | 37 |
| hsa05414 | Dilated cardiomyopathy | Human Diseases | 31/1428 | 0.0007754 | 0.01328569 | 31 |
| hsa05410 | Hypertrophic cardiomyopathy (HCM) | Human Diseases | 30/1428 | 0.0004615 | 0.0095375 | 30 |
| hsa05204 | Chemical carcinogenesis | Human Diseases | 29/1428 | 0.0006825 | 0.01322252 | 29 |
| hsa04512 | ECM-receptor interaction | Environmental Information Processing | 29/1428 | 0.0008571 | 0.01328569 | 29 |
| hsa04742 | Taste transduction | Organismal Systems | 29/1428 | 0.0008571 | 0.01328569 | 29 |
| hsa05140 | Leishmaniasis | Human Diseases | 28/1428 | 9.72E-05 | 0.00301393 | 28 |
| hsa04911 | Insulin secretion | Organismal Systems | 28/1428 | 0.0034847 | 0.04501106 | 28 |

**Table S3.** Clinicopathological characteristics of the CRC patients for snoRNA microarray.

| Patient | Patient's ID | Diagnosis | Pathology | Sex | Age | Weight (Kg) | Height (m) | TNM | Stage |
| --- | --- | --- | --- | --- | --- | --- | --- | --- | --- |
| #1 | 487416 | carcinoma of ascending colon | ulcerative adenocarcinoma | Male | 79 | 70 | 1.7 | T3N0M0 | IIA |
| #2 | 489005 | caicinoma of sigmoid colon | ulcerative adenocarcinoma | Male | 64 | 72 | 1.7 | T3N2aM0 | IIIB |
| #3 | 489180 | carcinoma of ileocecal colon | ulcerative adenocarcinoma | Female | 56 | 50 | 1.62 | T3N0M0 | IIA |

**Table S4.** Clinical information of the CRC patients.

| Patient's ID | Diagnosis | Pathology | Sex | Age | Weight (Kg) | Height (m) | TNM | Stage |
| --- | --- | --- | --- | --- | --- | --- | --- | --- |
| 489180 | carcinoma of ileocecal colon | ulcerative adenocarcinoma | Female | 56 | 50 | 1.62 | T3N0M0 | IIA |
| 488448 | carcinoma of ascending colon | bulging adenocarcinoma | Male | 77 | 77 | 1.72 | T4bN1aM1 | IVA |
| 486895 | carcinoma of sigmoid colon | ulcerative adenocarcinoma | Male | 62 | 80 | 1.64 | T3N0M0 | IIA |
| 488717 | carcinoma of sigmoid colon，carcinoma of transverse colon | bulging adenocarcinoma | Female | 66 | 54 | 1.55 | T3N1bM0 | IIIB |
| 488619 | carcinoma of ascending colon | ulcerative adenocarcinoma | Female | 37 | 46 | 1.64 | T3N0M0 | IIA |
| 487416 | carcinoma of ascending colon | ulcerative adenocarcinoma | Male | 79 | 70 | 1.7 | T3N0M0 | IIA |
| 488295 | carcinoma of ascending colon |  | Female | 64 |  |  |  |  |
| 488296 | carcinoma of ascending colon | ulcerative adenocarcinoma | Male | 49 | 83 | 1.8 | T4bN2aM1 | IV |
| 488096 | carcinoma of sigmoid colon | bulging adenocarcinoma | Female | 55 | 64 | 1.6 | T2N1bM0 | IIIA |
| 487329 | carcinoma of sigmoid colon | ulcerative adenocarcinoma | Male | 67 | 62 | 1.7 | T4bN2aM0 | IIIC |
| 489349 | carcinoma of ascending colon | ulcerative adenocarcinoma | Female | 65 | 67 | 1.69 | T3N2bM0 | IIIC |
| 487209 | carcinoma of ascending colon | ulcerative adenocarcinoma | Female | 50 | 51 | 1.61 | T3N0M0 | IIA |
| 489633 | carcinoma of ascending colon |  | Female | 69 | 55 | 1.55 |  |  |
| 489005 | carcinoma of sigmoid colon | ulcerative adenocarcinoma | Male | 64 | 72 | 1.7 | T3N2aM0 | IIIB |
| 489006 | carcinoma of ascending colon | ulcerative adenocarcinoma | Male | 38 | 67 | 1.75 | T3N2aM0 | IIIB |
| 488077 | carcinoma of sigmoid colon | bulging adenocarcinoma | Female | 76 | 63 | 1.6 | T3N0M0 | IIA |
| 488737 | carcinoma of colon | ulcerative adenocarcinoma | Female | 65 | 65 | 1.65 | T3N1aM0 | IIIB |
| 487254 | carcinoma of sigmoid colon | bulging adenocarcinoma | Female | 71 | 62 | 1.6 | T2N0M0 | I |
| 488669 | carcinoma of sigmoid colon | ulcerative adenocarcinoma | Female | 53 | 68 | 1.59 | T4aN2bM1 | IVA |
| 488856 | carcinoma of sigmoid colon | ulcerative adenocarcinoma | Male | 64 | 84 | 1.78 | T3N1aM0 | IIIB |
| 487067 | carcinoma of ascending colon | adenocarcinoma | Female | 52 | 57 | 1.6 | T3N1bM0 | IIIB |
| 487819 | carcinoma of sigmoid colon | ulcerative adenocarcinoma | Male | 48 | 89 | 1.78 | T2N0M0 | I |
| 478227 | carcinoma of sigmoid colon | ulcerative adenocarcinoma | Female | 51 | 57 | 1.64 | T4bN1aM1 | IV |
| 488551 | carcinoma of sigmoid colon | adenocarcinoma | Female | 74 | 44 | 1.56 | T3N0M0 | IIA |
| 526984 | rectal carcinoma | ulcerative adenocarcinoma | Female | 76 | 60 | 1.6 | T3N0M0 | IIA |
| 526754 | rectal carcinoma | ulcerative adenocarcinoma | Female | 60 | 62 | 1.62 | T3N1MO | IIIB |
| 526839 | rectal carcinoma | ulcerative adenocarcinoma | Male | 46 | 65 | 1.65 | T2N0M0 | I |
| 527300 | rectal carcinoma | ulcerative adenocarcinoma | Male | 71 | 72 | 1.65 | T2N0M0 | I |
| 527135 | carcinoma of transverse colon | ulcerative adenocarcinoma | Female | 65 | 60 | 1.62 | T4N1M0 | IIIB |
| 527185 | rectal carcinoma | ulcerative adenocarcinoma | Male | 52 | 74 | 1.75 | T3N0MO | IIA |
| 526214 | rectal carcinoma | bulging adenocarcinoma | Male | 53 | 70 | 1.76 | T2N0M0 | I |
| 526467 | rectal carcinoma, carcinoma of flexura coli dextra | ulcerative adenocarcinoma | Female | 78 | 58 | 1.6 | T3N0M0 | IIA |
| 526771 | carcinoma of ascending colon | adenocarcinoma | Male | 81 | 59 | 1.65 | T4N2bM0 | IIIC |
| 527549 | carcinoma of sigmoid colon | ulcerative adenocarcinoma | Male | 54 | 73 | 1.75 | T3N0M0 | IIA |
| 526459 | carcinoma of transverse colon | ulcerative adenocarcinoma | Male | 60 | 68 | 1.7 | T3N0M0 | IIA |
| 525661 | rectal carcinoma | ulcerative adenocarcinoma | Female | 72 | 40 | 1.6 | T3N1cM0 | IIIB |
| 526146 | rectal carcinoma | ulcerative adenocarcinoma | Male | 51 | 60 | 1.72 | T2N1aM0 | IIIA |
| 526311 | carcinoma of ascending colon | ulcerative adenocarcinoma | Male | 67 | 60 | 1.78 | T3N0M0 | IIA |
| 526073 | rectal carcinoma | ulcerative adenocarcinoma | Male | 67 | 45 | 1.69 | T3N0M0 | IIA |
| 525990 | carcinoma of descending colon | mucinous adenocarcinoma | Female | 49 | 65 | 1.63 | T4aN0M0 | IIB |
| 525744 | rectal carcinoma | ulcerative adenocarcinoma | Male | 53 | 71 | 1.6 | T3N0MO | IIA |
| 525565 | carcinoma of sigmoid colon | adenocarcinoma | Female | 41 | 63 | 1.58 | T3N0M0 | IIA |
| 525831 | carcinoma of sigmoid colon | adenocarcinoma | Female | 57 | 63 | 1.58 | T3N0M0 | IIA |
| 525615 | carcinoma of flexura coli dextra | bulging adenocarcinoma | Male | 55 | 64 | 1.73 | T3N0M0 | IIA |
| 525561 | carcinoma of sigmoid colon | ulcerative adenocarcinoma | Male | 61 | 65 | 1.7 | T4aN1bM0 | IIIB |
| 525658 | rectal carcinoma | ulcerative adenocarcinoma | Male | 63 | 74 | 1.76 | T3N0M0 | IIA |
| 525829 | carcinoma of flexura coli dextra | bulging adenocarcinoma | Female | 60 | 57 | 1.67 | T4aN1bM0 | IIIB |
| 525789 | rectal carcinoma | adenocarcinoma | Male | 57 | 78 | 1.8 | T3N0M0 | IIA |
| 525304 | carcinoma of sigmoid colon (with liver metastasis) | ulcerative adenocarcinoma | Female | 46 | 65 | 1.58 | T3N1aM1a | IVA |
| 525813 | carcinoma of transverse colon | adenocarcinoma | Female | 68 | 56 | 1.68 | T1N0M0 | I |
| 525298 | carcinoma of ascending colon | adenocarcinoma | Male | 74 | 69 | 1.73 | T2N0M0 | I |
| 525402 | rectal carcinoma | ulcerative adenocarcinoma | Male | 69 | 75 | 1.65 | T4aN2bM0 | IIIC |
| 524672 | rectal carcinoma | adenocarcinoma | Male | 71 | 55 | 1.75 | T3N1bM0 | IIIB |
| 527164 | rectal carcinoma | ulcerative adenocarcinoma | Female | 77 | 60 | 1.45 | T3N0M0 | IIA |
| 526332 | carcinoma of sigmoid colon | ulcerative adenocarcinoma | Male | 78 | 50 | 1.68 | T3N0M0 | IIA |
| 527139 | rectal carcinoma | ulcerative adenocarcinoma | Female | 69 | 62 | 1.57 | T3N0M0 | IIA |
| 527010 | rectal carcinoma | ulcerative adenocarcinoma | Male | 62 | 75 | 1.82 | T2N0M0 | I |

**Table S5.** List of primers and siRNAs used in this study.

| **Gene** | **Forward** | **Reverse** |
| --- | --- | --- |
| SNORA28 | ACACTCTGTGGCAGATGATCAAA | TGGGAGAGAGAACACCGGAAT |
| U6 | CTCGCTTCGGCAGCACA | AACGCTTCACGAATTTGCGT |
| SOCS1 | TTTTCGCCCTTAGCGTGAAGA | GAGGCAGTCGAAGCTCTCG |
| OSMR | ACTGGAACCTGCCACAGAGT | TCCAAGCTCACAATTCTCCA |
| LIFR | CATCATCAGCGTAGTGGCTAAA | TTCCGACCGAGACGAGTTA |
| IL-11 | CCCTGAAGACCCTGGAGCCCGAG | CACGGCCCAGTCAAGTGTCAGG |
| BRD4 | GAGCTACCCACAGAAGAAACC | GAGTCGATGCTTGAGTTGTGTT |
| GAPDH | CATGAGAAGTATGACAACAGCCT | AGTCCTTCCACGATACCAAAGT |
| **CUT&RUN Assay Primer** |  |  |
| LIFR#1 | ATC CCT TCC TTC AAT AAA TG | TGCTTC CAA AAT AGA GGA AC |
| LIFR#2 | GAT GAA TGG TAA GGG CGA TTT | CCC ACC TCC AGA GTT TCC A |
| LIFR#3 | GTG ATT TGG GTG CAA CTG G | TGA CTG GGA AGG GAA GAT GA |
| spike in | GCCTTCTTCCCATTTCTGATCC | CACGAATCAGCGGTAAAGGT |
| **Gene** | **Sense** | **Antisense** |
| JAK1 siRNA | GCACAGAAGACGGAGGAAAUGGUAU | AUACCAUUUCCUCCGUCUUCUGUGC |
| JAK2 siRNA | GGAUGGCAGUGUUAGAUAUTT | AUAUCUAACACUGCCAUCCTT |
| LIFR siRNA#1 | CCACACCGCUCAAAUGUUATT | UAACAUUUGAGCGGUGUGGTT |
| LIFR siRNA#2 | GAACAAAACGUUUCCUUAATT | UUAAGGAAACGUUUUGUUCTT |
| OSMR siRNA | CCAGAUCAGUAGGAUUGAATT | UUCAAUCCUACUGAUCUGGTT |
| BRD4 siRNA#1 | GAACCUCCCUGAUUACUAUTT | AUAGUAAUCAGGGAGGUUCTT |
| BRD4 siRNA#2 | UAAAUGAGCUACCCACAGATT | UCUGUGGGUAGCUCAUUUATT |
